# Situational enjoyment is associated with gaze behaviour during reading

**DOI:** 10.1038/s44271-026-00472-1

**Published:** 2026-05-13

**Authors:** Adam James Parker, Amrita Bains, Dorothy Zhiyu Gao, Emma Hance, Yunxi Li, Yifangjia Zhang

**Affiliations:** 1 Department of Experimental Psychology, Division of Psychology and Language Sciences, University College London, London, UK; 2 National Institute of Teaching, London, UK

**Keywords:** Human behaviour, Language and linguistics

## Abstract

Reading enjoyment is typically studied as a stable trait, yet enjoyment fluctuates within individuals. We examined how both trait- and state-level reading enjoyment are associated with engagement and eye movements during naturalistic reading. Seventy-six adults read 40 book synopses, rated their enjoyment, answered comprehension questions, and decided whether to wait to view the book cover, while their eye movements were recorded. Mixed-effects models disaggregated between-participant (trait) from within-participant (state) enjoyment. Both levels predicted greater willingness to wait and higher comprehension. Trait enjoyment was associated with longer passage and total word reading times. State enjoyment was linked to longer passage times, increased word skipping, faster early word processing, and increased regressions, suggesting more efficient first-pass reading accompanied by increased rereading. These findings suggest that momentary fluctuations in intrinsic motivation are systematically associated with differences in reading strategies, indicating a close link between perceived value and the allocation of cognitive resources during text processing.

## Abstract (web summary)

This study separates trait and state reading enjoyment, showing that both are associated with comprehension and willingness to wait to learn more about the text, but with distinct links to eye-movement behaviour.

## Introduction

Reading is central to success in literate societies, as it plays a crucial role in determining academic success, life outcomes, and well-being^1–4^. Yet reading is effortful, and we must make an active choice to pursue reading. Evidence suggests that there are clear benefits to being a motivated reader, with positive associations between reading motivation and vocabulary size, decoding ability, and comprehension^1,3,5^. Reading motivation is often measured at the trait level using self-report questionnaires. While incredibly useful, these measures are not suited to assess how motivation changes as readers encounter new texts across situations. The limited experimental work on situational motivation during reading has illustrated that the enjoyment of a text is associated with both reading comprehension and the willingness to incur a cost to find out more information about the material being read^6,7^. However, this work does not tell us how fluctuations in text enjoyment relate to processing in real time. This is the focus of the current research. Specifically, we examined how fluctuations in situational reading enjoyment were associated with engagement with text by implementing a willingness-to-wait paradigm while participants’ eye movements were recorded. In our analysis of the data, we disaggregated between-participant (trait) and within-participant (fluctuations in state) enjoyment, illustrating within a single study that both trait-level enjoyment and fluctuations in situational enjoyment were associated with readers’ eye movements in qualitatively different ways.

Traditionally, reading motivation is viewed as a relatively stable trait that reflects a person’s readiness to participate in reading activities^8^. Reading motivation is typically assessed through self-report questionnaires^9^, where researchers ask questions such as, *“Do you enjoy reading?”* Yet motivation is transient and affected by different factors, such as the purpose of the reading session, the different texts that are encountered, or people’s enjoyment during reading. To overcome the shortfalls of questionnaires, Bains et al.^6^ developed a willingness-to-wait task to examine the link between *liking* and *wanting* during reading, which are two components within Berridge and colleagues’^10–12^ neurobiological framework of reward, and have been used in prior work examining states of curiosity^13–15^. Participants read and rated forty book synopses for enjoyment. They were then asked whether they were willing to incur a time-related cost to see the book cover of the associated text before moving on to the next synopsis. The decision to wait was related to participants’ enjoyment of the synopsis, as was participants’ accuracy on comprehension questions, indicating that situation-specific reading enjoyment is related to both information seeking and understanding. Willingness-to-wait paradigms indicate that situational enjoyment is related to comprehension, raising an important question: *How is situational reading enjoyment related to how we interact with text?* One possibility is that higher enjoyment may be associated with deeper engagement, reflected in measurable differences in eye movements during reading.

The study of eye movements during reading has provided fine-grained insights into how readers coordinate attention and cognitive processing as they move both forwards and backwards through the text^16,17^. During reading, readers make a series of saccadic eye movements, separated by pauses known as fixations, during which information is encoded. Readers’ saccades and fixations can be used to calculate eye-movement measures. First-pass measures (e.g., word skipping, first-fixation duration, gaze duration) provide information about the early stages of lexical processing, such as word identification. Additional measures, such as regression likelihood, reflect the frequency of rereading, while measures like total reading time, which combine first-pass and subsequent fixations, capture processes of revision and reanalysis. Additionally, well-controlled eye-movement experiments have revealed that words that are more predictable from sentence context^18,19^, higher in their frequency of occurrence^20,21^, and shorter in length^22,23^ tend to have higher skipping rates and receive shorter fixation durations, highlighting that these words receive a processing advantage.

Research also indicates that task goals influence processing strategies that are captured in readers’ eye movements. For example, readers exhibit lower skipping rates, longer fixation durations, and more frequent regressions when reading for comprehension compared to skim reading^16,24^. Research also indicates that the depth of lexical processing is influenced by task goals: Kaakinen and Hyönä^25^ reported larger frequency effects when proofreading compared to reading for comprehension, and Schotter et al.^26^ reported larger predictability effects when proofreading required the successful integration of individual words with a sentence context. Regarding the relationship between reading motivation and readers’ eye movements, one study found that higher trait-level intrinsic motivation in children predicted shorter fixation durations and longer saccades, which are markers of more efficient processing, whereas extrinsic motivation showed reversed effects^27^. However, this study measured motivation via questionnaires and likely tapped into trait rather than state motivation. Additionally, they did not control for reading fluency, which is related to reading enjoyment^28,29^, and used an artificial moving-window paradigm^30,31^ where portions of isolated sentences were masked to the left and right of fixation, limiting ecological validity.

To date, the relationship between situational enjoyment and eye-movement behaviour during reading remains underexplored. If enjoyment does influence reading strategies in real time, this would provide be consistent with a dynamic link between situational intrinsic motivation and cognitive processing. The current study, therefore, employed the same willingness-to-wait paradigm as Bains et al.^6^, while also recording participants’ eye movements as they read book synopses. Participants rated their enjoyment of each synopsis before answering two comprehension questions. They then decided whether to incur a time-related cost to find out more information about a book or move on to the next synopsis. As such, we pre-registered two predictions based on Bains et al.’s results: (1) There will be a statistically reliable effect of reading enjoyment on decisions to wait, where greater enjoyment is associated with increased decisions to wait; and (2) There will be a statistically reliable effect of reading enjoyment on comprehension accuracy, where greater enjoyment is associated with increased comprehension accuracy.

We then conducted non-registered exploratory analyses of both global passage-level reading times and local word-level eye-movement measures, which were intended to examine how perceived intrinsic value is associated with moment-to-moment processes during reading. So that the relationship between reading enjoyment and outcomes was less likely to be attributable to baseline skill differences, we included reading fluency (measured via a sentence verification task) as a covariate in all analyses. Given the reported relationship between enjoyment and comprehension^6,7^, it is entirely possible that reading enjoyment would be associated with more regressive eye movements at the word-level, as regressions are tightly linked to comprehension^32^. Greater regressions could in turn coincide with more rereading and with longer word- and passage-level reading times. Alternatively, situational enjoyment could coincide with renewed information seeking and faster reading speed, resulting in shorter reading times at both the word- and passage-level; a pattern of results similar to work on questionnaire measures of enjoyment and eye-movement measures^27^. Observing either pattern of results would suggest that situational enjoyment is closely linked to the allocation of attention, language processing, and oculomotor control during reading, and would be consistent with the possibility that readers adjust processing in relation to perceived value. In addition, because situational enjoyment may be related to both eye-movement behaviour and to comprehension, we conducted a further exploratory analysis asking whether trial-level reading measures statistically accounted for the association between within-participant enjoyment and comprehension accuracy.

## Methods

### Transparency and openness

We report how we determined our sample size, all data exclusions, all manipulations, and all measures in the study. The confirmatory hypotheses, methods, and analyses were pre-registered on the Open Science Framework on the 15th October 2024: 10.17605/OSF.IO/J7N83. Deviations from the preregistration in the planned statistical analyses were made to maintain rigour, as detailed below. All book synopses and comprehension questions are publicly available: https://osf.io/2hx7w/files. Trial-level, word-level, and fixation-level reports are available as .csv files: https://osf.io/2hx7w/files. An R Markdown (.rmd) file is available to replicate all analyses and generate the original manuscript: https://osf.io/2hx7w/files.

### Participants

We pre-registered a minimum sample size of 40 participants. This minimum sample size was determined through a priori simulations reported by Bains et al.^6^. Based on an initial experiment with 40 participants, they reported that five participants would be required to achieve 90% power to detect an odds ratio of 6.57 (1.88 log-odds) at an alpha level of 0.05 for the analysis of decisions to wait. Bains et al. reported that 40 participants would be required to achieve 90% power to detect an odds ratio of 1.20 (0.18 log-odds) at an alpha level of 0.05 for the analysis of reading comprehension. However, we recruited 92 participants for the current study to provide a large enough sample for our exploratory analyses.

Participants were recruited via the University College London Psychology and Language Sciences SONA Participant Pool. Participants were aged between 18 and 40 years, had spoken English for a minimum of 10 years, had no language, hearing, or visual impairments, and had no history of neurological illness. Participants were reimbursed at a rate of £10.00/h or received course credit for their participation.

Of the 92 participants initially recruited, nine readers were excluded for incomplete data, and five were excluded due to excessive blinks and track loss during the eye-tracking portion of the experiment. As pre-registered, two additional participants were removed as they chose to wait on all trials during the willingness-to-wait task, indicating that they had potentially misunderstood the task. The final sample of 76 readers (60 females, 16 males, where gender was determined by self-identification) had a mean age of 22.18 (*SD*_*years*_ = 4.27). No data on socioeconomic status, communities of descent, or race and ethnicity were collected.

### Materials

Participants first completed two questionnaires: the Adult Reading Motivation Scale^33^, to assess reading motivation, and a reading engagement questionnaire. We had no a priori predictions regarding these questionnaire measures; however, given the ease of collecting this data, we decided to include both measures, should the data be useful in future exploratory work when generating novel hypotheses. These measures are, therefore, summarised in the Supplementary Methods. Participants then completed a sentence verification task, followed by a willingness-to-wait task while their eye movements were recorded.

### Sentence Verification Task

The Sentence Verification Task^34^ was included as a control measure to index individual differences in sentence-level reading fluency. Reading fluency was controlled for in all analyses to ensure that observed associations between reading enjoyment, comprehension, and eye-movement behaviour were not attributable to baseline differences in basic reading skills. The task included 80 sentences. Sentences reflected real-world knowledge and stayed on the screen for no more than 3 s. Participants had to indicate whether the sentences were true (e.g., *Bananas are fruit*) or false (e.g., *Cats have six legs*). Sentence length increased across blocks so that the task became more difficult. Participants had 90 s to respond to as many trials as possible. Participants received 1 point for each correct response, with a maximum possible score of 80.

### Willingness-to-wait task

#### Stimuli

Participants read 40 book synopses, taken from Bains et al.^6^. The synopses were taken verbatim from a popular online book merchant and included a variety of genres across fiction and non-fiction. On average, the synopses were 123.03 words long (*SD*_*words*_ = 30.02; *range*_*words*_: 63 – 182). The passages had a mean Flesch-Kincaid Grade Level of 11.77 (*SD*_*Flesch-Kincaid*_ = 4.44; *range*_*Flesch-Kincaid*_: 3.30–19.40). The mean word length was 4.83 letters (*SD*_*letters*_ = 2.71; *range*_*letters*_: 1–20). Words in each passage had an average Zipf frequency of 5.65 (*SD*_*zipf*_ = 1.51, *range*_*zipf*_: 1.17–7.67) based on the SUBTLEX-UK Corpus^35^.

#### Apparatus

Eye movements were monitored using an SR Research EyeLink 1000-Plus eye-tracker, sampling at 1000 Hz. Participants viewed the stimuli binocularly; however, only one eye was recorded. To reduce head movements, a chin-and-forehead rest was used. Stimuli were presented on a 23.8-inch Dell G2422HS LCD monitor with a resolution of 1920 × 1080 pixels. Text appeared in 18-point Courier New font, displayed as black text on a white background. The viewing distance was maintained at 84 cm, such that each character subtended 0.26° of visual angle horizontally. The experiment was created in SR Research Experiment Builder and administered on a Windows 11 computer.

#### Procedure

The eye-tracking procedure started with a 9-point calibration and validation procedure. Calibration accuracy was kept at <0.4° across the experiment. Drift checks were presented before every trial, and participants were recalibrated whenever necessary (and, at a minimum, every 10 trials). Participants were offered breaks whenever recalibration occurred. Participants completed one practice trial, followed by 40 experimental trials. The order of synopses was randomised for all but 10 participants, who took part in a version of the experiment where there was a coding error. As such, a sensitivity analysis was conducted excluding the 10 participants who received non-randomised allocation. The results were consistent with the primary analyses in terms of direction and statistical significance. Consequently, the full sample is retained in the main text to maximise statistical power.

Each trial started with a fixation point that appeared to the left of the first character on the first line. Once a stable fixation was detected, the experimenter started the trial. Participants then read a synopsis and pressed the *space bar* once they had finished reading. To judge familiarity, participants were asked whether they had read the book previously. Participants then rated how much they enjoyed reading the item on a scale from 1 (“hated it”) to 9 (“loved it”). Subsequently, participants answered two multiple-choice comprehension questions, one literal and one that involved drawing inferences. Participants were then given a choice to see the book cover of the synopsis they had read. They could either *skip* and move on to the next trial or *wait* to see the cover and find out more information about the book. If participants chose to *skip* the cover, they would wait 1–2 s before the next trial began. If participants chose to *wait*, they would wait 4–6 s before viewing the cover. During the willingness-to-wait task, responses were recorded via button responses on a keyboard.

### General procedure

Participants were tested in a laboratory room at University College London. Participants were first asked to read an information sheet before providing written informed consent. Participants completed the Adult Motivation Reading Scale^33^, followed by the Reading Engagement Questionnaire, Sentence Verification Task^34^, and the Willingness-to-Wait Task. The whole session lasted approximately 90 min. Participants were debriefed at the end of the experiment.

### Ethics

The experimental procedure was granted ethical approval by the UCL Department of Experimental Psychology’s Ethics Chair, ethics application number: EP/2021/015.

## Data analysis

### Data cleaning

We pre-registered that we would remove trials where participants had read the book previously. This led to the removal of 3.59% of trials. We additionally pre-registered that we would remove participants with less than 50% of trials following this cleaning step. However, no participants were removed for this reason. This left a total of 2931 trials for analysis.

### Confirmatory analyses

For the confirmatory analyses of decisions to wait and comprehension, we initially pre-registered a generalised linear mixed-effects analysis of trial-level data, where trial-level enjoyment ratings have been visualised in the Supplementary Pre-Registered Analyses. In these analyses, trial-level reading enjoyment would be used to predict each outcome measure. One drawback of this approach is that it cannot distinguish between-person (i.e., trait) and within-person (i.e., state-based situational/trial-level) reading enjoyment on the outcome measure. We, therefore, departed from our pre-registered analysis and opted for one that included both participant mean enjoyment ratings (one value per participant) and participant-mean centred enjoyment ratings, where a participant’s mean score is subtracted from the trial-level enjoyment (one value per participant per trial), as predictor variables. This enabled disaggregation of between- and within-person effects^36^ of reading enjoyment, where effects of the participant-mean centred score (situational enjoyment) reflect how trial-level deviations from a participant’s mean (trait-level) enjoyment score were associated with each outcome measure. This has been applied in many research areas, such as in research looking at enjoyment and learning strategies^37^, mindless reading^38^, and psychological well-being^39^. An advantage of characterising trait-level, between-participant, enjoyment based on data generated in the task is that it provides a direct measure of trait-level intrinsic motivation in context. At this point, it is important to note that there was no significant correlation between more general reading motivation scores on the Adult Reading Motivation Scale and between-participant enjoyment, *r*(74) = 0.16, *95% CI* [-0.07, 0.37], *p* = 0.169. Previously, Bains et al.^6^ also reported relatively weak correlations between trial-level enjoyment and the Adult Reading Motivation Scale, *r* = 0.27. Together, these findings suggest that behavioural measures of enjoyment during reading capture affective responses that are only partially related to broader, self-reported motivational dispositions. Importantly, the between-participant enjoyment measure used here reflects stable individual differences in situational enjoyment across trials, rather than global trait-level reading motivation. For simplicity in the current work, we refer to the participant mean score as *between-participant enjoyment* and the participant-mean centred score as *within-participant enjoyment*. We reported the full pre-registered analyses in the Supplementary Pre-Registered Analyses.

Generalised linear mixed-effects models were fitted to analyse binary decision to wait and comprehension accuracy data using the *glmer()* function from the lme4 package^40^ [version 1.1.37] within R^41^ [version 4.5.0]. The model fitted to decision to wait data initially adopted the structure: *dv ~ between-participant enjoyment + within-participant enjoyment* + *(1 + within-participant enjoyment|participant)* + *(1 +  between-participant enjoyment + within-participant enjoyment|item)*, where participant and item are random factors. For the analysis of comprehension accuracy, the model initially adopted the structure: *dv ~ between-participant enjoyment + within-participant enjoyment + reading fluency* + *(1 + within-participant enjoyment|participant)* + *(1 + between-participant enjoyment + within-participant enjoyment|item)*. Here, reading fluency, as indexed by sentence verification score, was included as a covariate as it was significantly correlated with comprehension scores, *r*(74) = 0.59, *95% CI* = [0.42, 0.72], *p* < 0.001. Reading fluency was centred prior to analysis. To determine the optimal random-effects structure for each model, we used the *buildmer()* function from the buildmer package^42^ [version 2.12]. Initially, we fitted a maximal random-effects structure to the data. We then implemented a backward selection procedure, sequentially removing terms based on the significance of changes in model log-likelihood between successive models. To check for the robustness of our results of *buildmer()* models, we refitted the models following the principled “keep it maximal” recommendations reported by Barr et al.^43^, with the fully random effects structure justified by the experimental design. We then implemented a principled, step-wise simplification procedure to determine the final random effect structure. Ultimately, this different approach yielded the same pattern of results as the models reported in the main text and our conclusions remained unchanged. As such, we report the the final *buildmer()* models. For each reported model, we confirmed that the assumptions of statistical tests had been met by using the *check_model()* function from the performance package^44^ [version 0.15.2], an observation that also held for all exploratory linear mixed-effects models. For all models (confirmatory and exploratory), we report regression coefficients (*b*), standard errors (*SE*), 95% confidence intervals (*95% CI*), *t/z*-values, where |*t* | > 1.96 and |*z* |  >1.96 indicate a statistically significant fixed effect, and *p*-values.

### Exploratory analyses

#### Mixed-effect analyses of eye-movement measures

We conducted exploratory analyses to examine the effect of reading enjoyment on both global (passage-level) and local (word-level) measures. Prior to analysis, eye movement data were pre-processed using DataViewer (SR Research) to conduct vertical adjustment of fixations to the correct line. We then used the clean function within DataViewer to trim the data, where fixations shorter than 80 ms that were located within one-character space of the next or previous fixation were merged into that nearby fixation. Other fixations shorter than 80 ms or greater than 1200 ms were removed. We then removed observations from our dataset if they were preceded or followed by a blink. This resulted in 210,277 observations from 2910 trials across 76 participants, comprising 1819 unique word tokens for analysis.

#### Global reading measures

In an exploratory analysis, we fitted a linear mixed-effects model to passage reading time data. In this model, we controlled for reading fluency, passage difficulty (the Flesch-Kincaid grade level), and passage length (the number of words within a passage), where all covariates were centred prior to analysis. The model initially adopted the structure: *dv ~ between-participant enjoyment + within-participant enjoyment + reading fluency + passage difficulty + passage length* + *(1 + within-participant enjoyment|participant)* + *(1 + between − participant enjoyment + within-participant enjoyment|item)*. The best-fitting random effects structure was determined via the *buildmer()* function within R.

#### Local reading measures

To examine how reading enjoyment was associated with the moment-to-moment decisions made during reading, we analysed five eye-movement measures: word skipping (the likelihood that a word is not fixated during a first-pass), first-fixation duration (the duration of the very first fixation on a word during a first-pass), gaze duration (the sum of all first-pass fixations on a word before leaving that region), regressions out (the likelihood of making a regression out of a word), and total reading time (the sum of all fixations on a word irrespective of when they occurred). In these (generalised) linear mixed-effects analyses, we controlled for reading fluency, Zipf frequency, and word length. The model initially adopted the structure: *dv ~ between-participant enjoyment + within-participant enjoyment + reading fluency + frequency + length* + *(1 +  within-participant enjoyment|participant)* + *(1 + between-participant enjoyment + within-participant enjoyment|item)* + *(1 + between-participant enjoyment + within-participant enjoyment|word)*. Again, the best-fitting random effects structure was determined via the *buildmer()* function within R.

#### Mediation analyses of trial-level data

Following our linear mixed-effects analyses, we conducted further exploratory mediation analyses for eye-movement measures that were associated with within-participant enjoyment to examine whether aggregated trial-level reading measures statistically accounted for part of the association between situational enjoyment and comprehension accuracy. For each reading measure, we fitted joint two-equation Bayesian multi-level models using the *brm* function from the brms package^45^ [version 2.22.0]. This approach preserves the crossed random-effects structure of the data and allows indirect effects to be derived directly from posterior draws, ensuring that uncertainty in both paths is fully reflected in the mediation estimates. The first submodel predicted the trial-level mediator (e.g., proportion of skipping, average first-fixation duration) using the model: *dv ~ between-participant enjoyment + within-participant enjoyment + reading fluency + passage difficulty + passage length* + *(1 + within-participant enjoyment|participant)* + *(1 + between-participant enjoyment + within-participant enjoyment|item)*. The second submodel predicted comprehension accuracy using the model: *dv ~ between-participant enjoyment + within-participant enjoyment + mediator + reading fluency + passage difficulty + passage length* + *(1 + within-participant enjoyment|participant)* + *(1 + between-participant enjoyment + within-participant enjoyment|item)*. The data met the assumptions of these models. Models were estimated using default priors, 4000 iterations, with four chains, where the first 2000 were discarded due to warm-up. Direct paths were estimated using the coefficient for the enjoyment-comprehension path. Indirect effects were computed as the product of posterior draws for the enjoyment-mediator path (a) and the mediator-comprehension path (b). For all direct and indirect effects, we report posterior means (b) and 95% credibility intervals (95% CrI). Using these results, effects can be interpreted as statistically reliable when the credibility intervals exclude zero. If both the direct and indirect paths were reliable, then this would indicate partial mediation. If the indirect path but not the direct path were reliable, then it would indicate full mediation.

## Results

### Confirmatory analyses

**Fig. 1:**
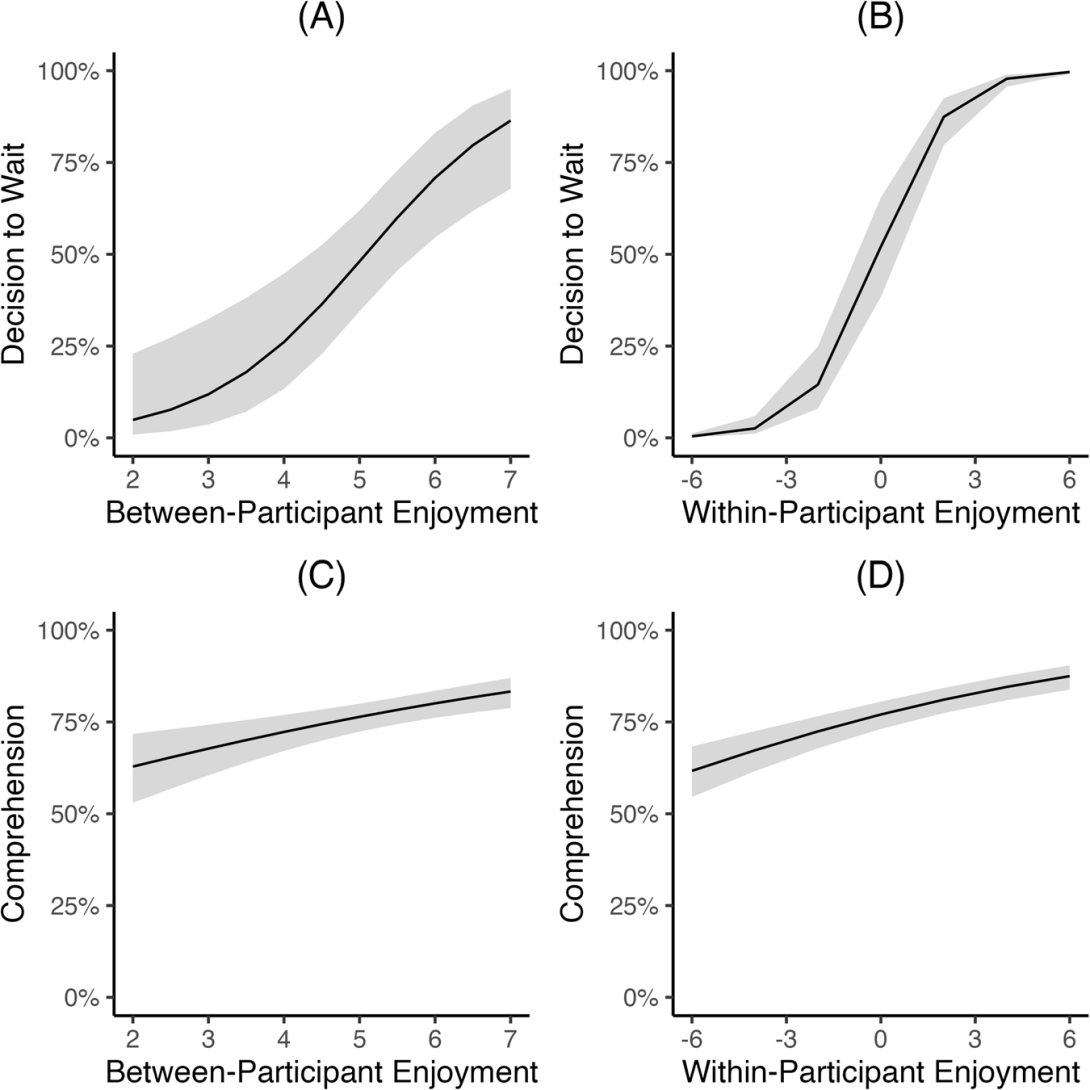
The Effect of Between-Participant and Within-Participant Enjoyment on Decision to Wait and Comprehension. Note. The relationship between (**A**) between-participant reading enjoyment and decisions to wait, (**B**) within-participant reading enjoyment and decisions to wait, (**C**) between-participant reading enjoyment and comprehension accuracy, and (**D**) within-participant reading enjoyment and comprehension accuracy. Solid lines represent model-predicted probabilities from generalised linear mixed-effects models. Shaded bands indicate 95% confidence intervals around the fixed-effect estimates. Generalised linear mixed-effects models were fitted to 2,931 observations for the decision-to-wait analysis and 5,862 observations for the comprehension-accuracy analysis.

The model fitted to binary decision to wait data (*glmer(dv ~ between-participant enjoyment + within-participant enjoyment* + *(1 + within-participant enjoyment|participant)* + *(1|item))*; 2931 observations) indicated that both between-participant enjoyment and within-participant enjoyment were significant predictors of the likelihood of waiting to see the cover of a book (see Table 1), where a one-unit increase in between-participant enjoyment was associated with a 2.62-fold increase in the odds of making a decision to wait. That is, participants with higher mean enjoyment ratings (i.e., higher between-participant enjoyment) were more likely to wait to see the cover of a book (see Fig. 1). Furthermore, as enjoyment at the trial level increased, relative to a participant’s mean enjoyment rating, participants were more likely to wait to see the cover of a book. A one-unit increase in within-participant enjoyment was associated with a 2.53-fold increase in the odds of making a decision to wait. This indicates that both trait-based and situational reading enjoyment are associated with decisions to wait.

**Table 1:** Generalized linear mixed-effects results for decision to wait and comprehension

Measure	Fixed Effect	*b*	SE	95% CI	*z*-value	*p* value
Decision to Wait	(Intercept)	−4.896	1.427	−7.694, −2.099	−3.43	<0.001
	Between-Participant Enjoyment	0.964	0.270	0.434, 1.493	3.57	<0.001
	Within-Participant Enjoyment	0.929	0.075	0.781, 1.076	12.36	<0.001
Comprehension	(Intercept)	0.062	0.307	-0.540, 0.664	0.20	0.839
	Between-Participant Enjoyment	0.216	0.057	0.105, 0.327	3.82	<0.001
	Within-Participant Enjoyment	0.122	0.018	0.087, 0.157	6.86	<0.001
	Reading Fluency	0.029	0.006	0.016, 0.041	4.51	<0.001

The model fitted to binary comprehension accuracy data (*glmer(dv ~ between-participant enjoyment + within-participant enjoyment + reading fluency* + *(1|participant)* + *(1|item))*; 5862 observations) indicated that both between-participant enjoyment and within-participant enjoyment were significant predictors of the likelihood of answering a comprehension question correctly. A one-unit increase in between-participant enjoyment was associated with a 1.24-fold increase in the odds of answering a comprehension question correctly, indicating that participants with higher mean enjoyment ratings were more likely to answer a question correctly. Furthermore, a one-unit increase in within-participant enjoyment was associated with a 1.13-fold increase in the odds of answering a comprehension question correctly, indicating that as enjoyment at the trial level increased, relative to a participant’s mean enjoyment rating, participants were more likely to answer a comprehension question correctly.

### Exploratory analyses

#### Mixed-effect analyses of eye-movement measures

##### Global reading measures

**Fig. 2:**
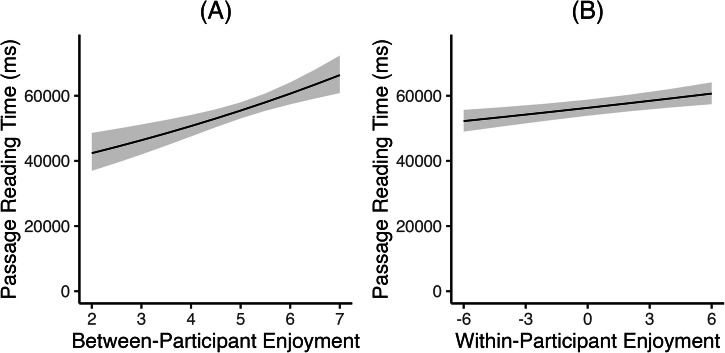
The Effect of Between-Participant and Within-Participant Enjoyment on Passage-Level Reading Time. Note. The relationship between (**A**) between-participant reading enjoyment and passage reading time, and (**B**) within-participant reading enjoyment and passage reading time. Solid lines represent model-predicted values from linear mixed-effects models fitted to log-transformed passage durations. Shaded bands indicate 95% confidence intervals around the fixed-effect estimates. Linear mixed-effects models were fitted to 2931 observations.

The model fitted to log-transformed reading time data (*lmer(dv ~ between-participant enjoyment + within-participant enjoyment + reading fluency + passage difficulty + passage length* + *(1 + within-participant enjoyment|participant)* + *(1 + within-participant enjoyment|item))*; 2931 observations) indicated that passage reading time increased as a function of between-participant enjoyment and within-participant enjoyment (Fig. 2), where a one-unit change in between-participant enjoyment resulted in a 9.38% increase in passage reading time. As such, participants with higher mean enjoyment ratings had longer passage reading times (Table 2). Furthermore, as participants’ enjoyment ratings increased, relative to their mean enjoyment rating, passage reading times also increased, where a one-unit change in within-participant enjoyment resulted in a 1.26% increase in passage reading time. This indicates that both measures of reading enjoyment are associated with longer reading times when controlling for reading fluency, passage length, and passage difficulty.

**Table 2:** Linear mixed-effects results for passage reading time

Measure	Fixed Effect	*b*	SE	95% CI	*t*-value	*p* value
Passage Reading Time	(Intercept)	10.485	0.109	10.272, 10.699	96.10	<0.001
	Between-Participant Enjoyment	0.090	0.021	0.049, 0.130	4.30	<0.001
	Within-Participant Enjoyment	0.012	0.003	0.006, 0.019	3.64	<0.001
	Reading Fluency	−0.010	0.002	−0.014, −0.005	−4.14	<0.001
	Passage Difficulty	0.008	0.002	0.003, 0.012	3.12	0.004
	Passage Length	0.004	<0.001	0.004, 0.005	12.20	<0.001

##### Local reading measures

**Fig. 3:**
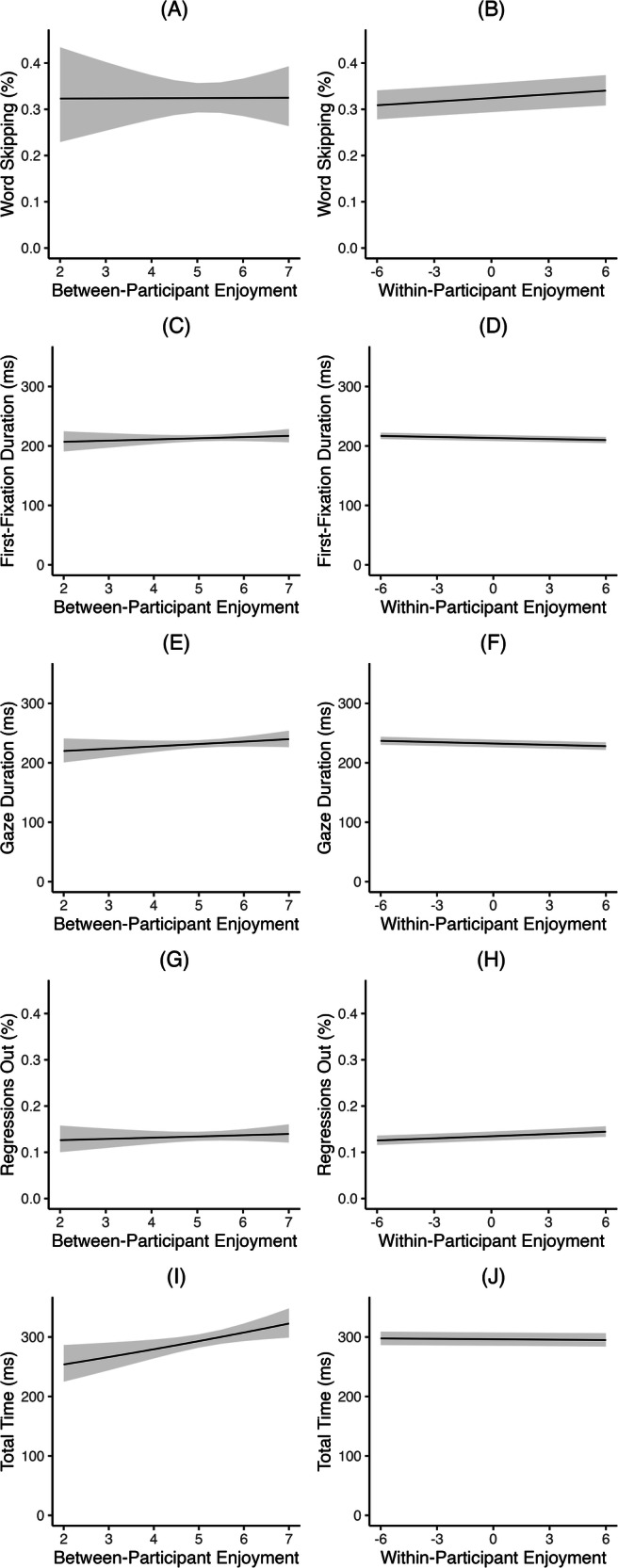
The Effect of Between-Participant and Within-Participant Enjoyment on Word-Level Measures. Note. The relationship between (**A**) between-participant reading enjoyment and word skipping, (**B**) within-participant reading enjoyment and word skipping, (**C**) between-participant reading enjoyment and first-fixation duration, (**D**) within-participant reading enjoyment and first-fixation duration, (**E**) between-participant reading enjoyment and gaze duration, (**F**) within-participant reading enjoyment and gaze duration, (**G**) between-participant reading enjoyment and regressions out, (**H**) within-participant reading enjoyment and regressions out, (**I**) between-participant reading enjoyment and total reading time, and (**J**) within-participant reading enjoyment and total reading time. Solid lines represent model-predicted values from generalised linear mixed-effects models (for skipping and regressions out) and linear mixed-effects models fitted to log-transformed reading times (for first-fixation duration, gaze duration, and total reading time). Shaded bands indicate 95% confidence intervals around the fixed-effect estimates. Generalised linear mixed-effects models were fitted to 210,277 observations for skipping and regressions out. Linear mixed-effects models were fitted to 139,680 observations for first-fixation duration and gaze duration, and 201,277 observations for total reading time.

The model fitted to skipping likelihood data (*glmer(dv ~ between-participant enjoyment + within-participant enjoyment + reading fluency + frequency + length* + *(1|participant)* + *(1|item))*; 210,277 observations) indicated that word skipping significantly increased with increasing within-participant enjoyment (see Table 3). A one-unit increase in within-participant enjoyment was associated with a 1.01-fold increase in the odds of skipping a word (see Fig. 3).

**Table 3:** (Generalized) Linear mixed-effects results for word-level measures

Measure	Fixed effect	*b*	SE	95% CI	*z*/*t*-value	*p* value
Skipping Likelihood	(Intercept)	−0.751	0.362	−1.461, −0.041	−2.07	0.038
	Between-Participant Enjoyment	0.002	0.069	−0.134, 0.138	0.02	0.982
	Within-Participant Enjoyment	0.012	0.003	0.007, 0.017	4.37	<0.001
	Reading Fluency	0.011	0.008	−0.005, 0.026	1.35	0.177
	Zipf Frequency	0.021	0.005	0.011, 0.031	4.24	<0.001
	Word Length	−0.131	0.003	−0.136, −0.125	−46.15	<0.001
First-Fixation Duration	(Intercept)	5.314	0.066	5.184, 5.443	80.5	<0.001
	Between-Participant Enjoyment	0.009	0.013	-0.015, 0.034	0.75	0.457
	Within-Participant Enjoyment	−0.003	<0.001	−0.004, -0.002	−5.64	<0.001
	Reading Fluency	−0.006	0.001	−0.009, −0.003	−4.44	<0.001
	Zipf Frequency	−0.031	0.002	−0.036, −0.027	−14.05	< 0.001
	Word Length	−0.001	0.001	−0.003, 0.001	-0.86	0.389
Gaze Duration	(Intercept)	5.350	0.074	5.205, 5.494	72.59	<0.001
	Between-Participant Enjoyment	0.017	0.014	−0.010, 0.045	1.23	0.222
	Within-Participant Enjoyment	−0.003	0.001	−0.004, −0.002	-5.65	<0.001
	Reading Fluency	−0.009	0.002	−0.012, −0.006	-5.77	<0.001
	Zipf Frequency	−0.059	0.003	-0.065, -0.053	-19.89	<0.001
	Word Length	0.018	0.001	0.015, 0.020	13.51	<0.001
Regression Out Likelihood	(Intercept)	−1.979	0.204	−2.379, −1.579	−9.70	<0.001
	Between-Participant Enjoyment	0.023	0.039	−0.053, 0.099	0.60	0.551
	Within-Participant Enjoyment	0.013	0.003	0.007, 0.020	4.08	< 0.001
	Reading Fluency	−0.003	0.004	−0.011, 0.005	−0.69	0.49
	Zipf Frequency	0.048	0.019	0.011, 0.085	2.55	0.011
	Word Length	0.092	0.008	0.075, 0.109	10.93	<0.001
Total Reading Time	(Intercept)	5.448	0.097	5.258, 5.638	56.15	<0.001
	Between-Participant Enjoyment	0.048	0.019	0.012, 0.084	2.59	0.012
	Within-Participant Enjoyment	−0.001	0.001	−0.002, <0.001	−1.16	0.246
	Reading Fluency	−0.008	0.002	−0.012, −0.004	−3.73	<0.001
	Zipf Frequency	−0.102	0.004	−0.111, −0.094	−24.30	<0.001
	Word Length	0.025	0.002	0.022, 0.029	13.77	<0.001

For first-fixation duration and gaze duration, which are considered to be early measures of word processing, the models (*lmer(dv ~ between-participant enjoyment + within-participant enjoyment + reading fluency + frequency + length* + *(1|participant)* + *(1|item)* + *(1|word))*; 139,680 observations) indicated there was a statistically significant effect of within-participant enjoyment on each measure. A one-unit change in within-participant enjoyment resulted in a 0.27% decrease in first-fixation duration and a 0.32% decrease in gaze duration. This indicates that participants’ initial reading times decreased as trial-level enjoyment increased relative to their mean enjoyment rating.

The model fitted to regression out likelihoods (*glmer(dv ~ between-participant enjoyment + within-participant enjoyment + reading fluency + frequency + length* + *(1|participant)* + *(1|word))*; 210,277 observations) indicated that the likelihood of making a regression increased with increasing within-participant enjoyment. A one-unit increase in within-participant enjoyment was associated with a 1.01-fold increase in the odds of making a regression. The estimates for between-participant enjoyment in the models predicting skipping likelihood, first-fixation duration, gaze duration, and regression-out likelihood did not reach statistical significance.

For total reading time, the model fitted to total reading times (*lmer(dv ~ between-participant enjoyment + within-participant enjoyment + reading fluency + frequency + length* + *(1|participant)* + *(1|item)* + *(1|word))*; 210,277 observations) indicated that between-participant enjoyment significantly predicted total reading times on words, where a one-unit change in between-participant enjoyment was associated with a 4.91% increase in total reading time. This indicates that participants with higher enjoyment ratings overall spent longer on individual words. The estimate for within-participant enjoyment did not reach statistical significance for total reading time.

##### Mediation analyses of trial-level data

For eye-movement measures that were associated with within-participant enjoyment, we next conducted exploratory mediation analyses to test whether the trial-level reading measures statistically accounted for the association between within-participant enjoyment and comprehension. In the analysis examining passage reading time as a mediator of the association between within-participant enjoyment and comprehension, the direct path from within-participant enjoyment to comprehension was statistically reliable, *b* = 0.13, *95% CrI* [0.08, 0.17]. In contrast, the indirect path via passage reading time was not statistically reliable, *b* = −2.74e-03, *95% CrI* [−8.05e-03, 1.37e-03]. The next analysis examined whether the proportion of words skipped on a trial mediated the association between within-participant enjoyment and comprehension. Again, the direct path was statistically reliable, *b* = 0.12, *95% CrI* [0.08, 0.17], but the indirect path was not, *b* = −3.77e-05, *95% CrI* [−1.88e-03, 1.80e-03]. The mediation analysis examining whether the mean first-fixation duration on a trial mediated the association between within-participant enjoyment and comprehension again yielded a reliable direct path, *b* = 0.12, *95% CrI* [0.08, 0.17], but no statistically reliable indirect path, *b* = 7.87e-04, *95% CrI* [−2.28e-03, 4.17e-03]. The corresponding analysis for mean gaze duration showed the same pattern, with a reliable direct path, *b* = 0.12, *95% CrI* [0.08, 0.16], and a non-reliable indirect path, *b* = 1.24e-03, *95% CrI* [−1.78e-03, 4.85e-03]. Finally, the mediation analysis of trial-level regressions revealed a statistically reliable direct path, *b* = 0.12, *95% CrI* [0.08, 0.16], and a non-reliable indirect path, *b* = 4.13e-04, *95% CrI* [−9.19e-04, 2.51e-03]. Taken together, these mediation analyses showed that the indirect effects via the aggregated trial-level reading measures were not statistically reliable, whereas the direct effect of within-participant enjoyment on comprehension remained reliable.

## Discussion

We set out to examine how fluctuations in reading enjoyment are associated with text processing by recording participants’ eye movements as they read book synopses and completed a willingness-to-wait task. By disaggregating between-participant (trait) and within-participant (state) enjoyment, we show that both stable trait-like enjoyment and momentary fluctuations predicted a greater willingness to incur a cost to learn more about a text and higher comprehension accuracy. Again, both levels of enjoyment were linked to longer passage reading times. While higher trait-level enjoyment was linked to longer total reading times on words, increases in situational enjoyment were associated with increased word skipping, shorter early first-fixation and gaze durations, and more frequent regressions. The mixed-effects analyses suggest that enjoyment is associated with readers’ eye-movement behaviour, raising the possibility that reading behaviour may relate to comprehension. To test these potential mediating effects, we conducted exploratory mediation analyses using aggregated trial-level variables. These revealed statistically reliable direct paths between situational enjoyment and comprehension in the models, but no reliable indirect effects via eye-movement measures. Accordingly, these exploratory analyses suggest that the association between situational enjoyment and comprehension remained after accounting for the aggregated trial-level reading measures examined here, rather than being statistically accounted for by them. Together, these results suggest that fluctuations in reading enjoyment are associated with the allocation of attention and cognitive resources during reading relatively independently of comprehension.

We are not the first to suggest that situational reading enjoyment is related to the decision to incur a cost to find out more information about a text and increased reading comprehension^6,7^. However, by disaggregating between-participant and within-participant enjoyment^36^, we provide stronger evidence that situational enjoyment is associated with both wanting and understanding during reading. Positive shifts away from a participant’s mean enjoyment rating, where trials were more enjoyable, predicted increased decisions to wait and higher comprehension accuracy. Because within-participant enjoyment is statistically independent of overall enjoyment, these results hold even for the least motivated readers.

Previously conducted work on situational reading enjoyment has focused on how enjoyment relates behavioural choices and comprehension. In addition to offline, end-state decisions, the current work examined how fluctuations in situational reading enjoyment were associated with processing during “online” text processing. We originally considered two ways in which situational enjoyment might be associated with processing. First, given prior links between enjoyment and comprehension, higher enjoyment might be associated with longer reading times, more regressions, and more rereading, alongside higher comprehension. Second, increases in enjoyment might coincide with renewed information seeking and faster reading speed, resulting in shorter reading times. Indeed, fluctuations in reading enjoyment were associated with differences in eye-movement behaviour. However, the results observed did not neatly map onto either shorter or longer reading times under high enjoyment. Instead, when enjoyment for a given passage exceeded a reader’s own average enjoyment, early fixation measures (first-fixation duration and gaze duration) were shorter. At the same time, positive fluctuations in enjoyment predicted increased skipping and were also associated with more regressions back through the text. These regressions coincided with longer passage-level reading times; descriptively, this corresponded to an approximately 9-s difference between the least and most enjoyed passages. This dynamic modulation of eye movements resembles evidence that lexical processing is shaped by task goals^25,26^. Importantly, the pattern we observed was associated with internal states of intrinsic motivation, in the absence of explicit task goals.

Observing a pattern of rapid initial uptake, combined with more frequent rereading, may suggest that enjoyment is associated with both greater efficiency in early word recognition and greater investment in higher-order integration processes that support comprehension^32^. To test whether the association between enjoyment and comprehension might be statistically accounted for by reading strategies that are measurable using eye-movement measures (particularly regressions and total passage reading time), we conducted mediation analyses estimating both the direct effect of enjoyment on comprehension and the indirect effect via eye-movement measures. These Bayesian analyses yielded evidence for a reliable direct association between enjoyment and comprehension but no indirect effects via eye movements. As such, based upon the available evidence, the eye-movement measures examined here are unlikely to statistically account for the association between enjoyment and comprehension.

So why might fluctuations in situational reading enjoyment be linked to eye-movement measures? Our data do not support a comprehension-based account. An alternative possibility, then, may be that enjoyment and readers’ eye movements are a product of reading proficiency. If the association were driven solely by processing fluency or other trait-like skill differences, effects should have been linked (to some extent) to between-participant enjoyment. Instead, systematic shifts in early fixation durations, skipping, and regressions were uniquely associated with momentary fluctuations in enjoyment relative to a reader’s mean enjoyment. This pattern does not establish that enjoyment causally shapes processing; however, it constrains purely fluency-based explanations and is more consistent with accounts in which perceived value and cognitive processing are closely coupled during reading.

A further possibility is that increases in enjoyment coincide with new cycles of information seeking and with differences in how readers engage with text in real time. Indeed, the Situated Expectancy-Value Theory^46,47^ suggests that intrinsic value fluctuates with situational context and guides behavioural choices. In practice, such enjoyment may manifest as a drive for anticipatory prediction, enabling readers to streamline processing and navigate the text with greater speed to gain new information. Under a Predictive Processing framework^48^, increased enjoyment may be associated with greater engagement in active lexical prediction for upcoming words. This could reduce the need for extended visual sampling, a suggestion that is consistent with increased skipping and shorter fixation times^18,19^. Simultaneously, this quicker initial processing may heighten the need to monitor coherence. Within this framework, readers generate weighted probabilistic expectations about upcoming input. While strong predictions facilitate rapid uptake, any deviations generate a “prediction error”. This error may prompt strategic rereading and regressions to restore coherence. When comprehension breaks down, readers who are enjoying the text more may be more likely to utilise these regressive eye movements to resolve inconsistency. This suggested use of predictive processing aligns with the later stages of the Four-Phase Model of Interest Development^49^, which characterises developed interest by the ability to anticipate subsequent processing steps and utilise self-regulation to navigate complex or unexpected content. Consequently, the observed balance between efficiency and integration may reflect a state in which readers appear more engaged in seeking and resolving information to satisfy an emerging or well-developed interest.

An alternative account of the current results comes from Cognitive Load Theory^50,51^. Positive affect has been shown to reduce perceived cognitive load and free working memory resources^52^, which may allow readers to allocate attention more flexibly toward higher-order integration processes. Under such an account, shorter early fixations and increased regressions need not reflect stronger predictions per se, but rather a temporary expansion of available cognitive resources that supports both efficient word recognition and deeper coherence monitoring. Based on the current data, it is difficult to tease apart the exact mechanism underlying these enjoyment effects. It will, therefore, be down to future studies to test whether predictive processing, cognitive load, and other accounts drive these within-participant enjoyment effects.

Regardless, it is important to consider the practical significance of our results and any wider implications. Although the effects were modest in magnitude, this is not unexpected given the many factors that shape naturalistic reading. Their practical significance may therefore be incremental rather than large in isolation, but they nonetheless suggest that enjoyment is one factor associated with how readers allocate cognitive resources during comprehension. It is possible that such modest associations become more consequential over longer texts, with enjoyment potentially relating to more sustained reading and better comprehension. If intrinsic motivation is associated with greater investment in higher-order integration processes, then this work may have implications for intervention. Approaches that increase perceived value (e.g., by providing choice^53^ or allowing engagement with personally relevant material) may be relevant to how readers allocate cognitive resources during comprehension. However, this account assumes that enjoyment shapes reading behaviour. A limitation of the current study is its correlational design. We cannot determine whether enjoyment precedes and influences eye-movement behaviour, or whether successful processing enhances comprehension and thereby increases enjoyment. Supporting the latter possibility, Bains and colleagues^54^ found that successfully learning new words and extracting meaning from context is associated with greater enjoyment. Future research will need to disentangle whether enjoyment contributes to processing efficiency, reflects it, or both.

The disaggregation method employed also enables us to comment on more stable aspects of enjoyment and text processing. We report that between-participant, trait-level enjoyment results in longer overall reading times on both words and passages. The observation that participants with higher between-participant enjoyment ratings spent approximately 24 s longer on passages overall may suggest that these readers were more willing to invest time in processing the text before deciding to see a book cover, which ultimately resulted in better reading comprehension. Notably, this was observed even when there was no extrinsic reward in the task. This finding contrasts with prior research on children, which reported that intrinsic motivation (operationalised as enjoyment) was associated with faster text processing^27^. Perhaps this discrepancy reflects developmental differences in adults and children or different aspects of enjoyment measured in “online” paradigms and questionnaires.

### Limitations

Several limitations should be noted. Although we report statistically reliable associations between situational enjoyment, information seeking, and eye-movement behaviour, the data remain correlational. Consequently, fluctuations in enjoyment may shape and be shaped by how readers engage with the text. Future studies assessing directionality will require experimental manipulations of perceived value, through mechanisms such as providing choice or increasing affect, in combination with high-resolution eye-tracking to examine resulting changes in moment-to-moment processing. Such experimental studies may also be helpful in establishing whether the relatively modest effect sizes at the word-level reflect subtle but cumulative influences on processing or context-specific effects that emerge more strongly under certain conditions.

In addition, enjoyment was indexed once per text following reading, which constrains inferences about the temporal dynamics of intrinsic value during processing. Because enjoyment was not measured continuously, we cannot determine how enjoyment fluctuates within passages or at what point during reading intrinsic value begins to influence eye-movement control. This limitation is particularly relevant given that the materials consisted of relatively short synopses. Motivational dynamics may unfold differently during extended, self-directed reading, where sustained goals, fatigue, and strategic regulation operate over longer timescales. Replication using longer texts will be important for establishing when and how enjoyment influences processing.

Finally, text-level characteristics may plausibly contribute to the observed within-participant effects. A substantial body of research demonstrates that eye-movement patterns vary systematically as a function of text genre, with narrative texts often eliciting more linear progression and expository or reflective texts prompting increased rereading and regressions^55–59^. Because genre is linked to readers’ subjective experience, including enjoyment, variation across passages could contribute to the association between enjoyment ratings and eye-movement behaviour. The stimulus set spanned multiple genres with an uneven distribution (see Supplementary Exploratory Analyses of Text Properties), making this a reasonable concern. However, when we included a fixed effect coding for fiction versus non-fiction in the *Supplementary Pre-Registered Analyses*, the within-participant enjoyment effects remained statistically reliable across models. This pattern suggests that the observed associations are unlikely to reflect broad genre-based differences. Future research should manipulate genre and other text-based properties alongside intrinsic value to disentangle motivational effects from textual features.

## Conclusion

We set out to examine whether situational reading enjoyment is associated with the moment-to-moment decisions made by readers. By disaggregating within- and between-participant enjoyment, we were able to examine situational fluctuations more precisely and distinguish them from more stable, trait-like enjoyment, clarifying how each relates to reading behaviour. The results indicate that readers with higher trait-level enjoyment tend to spend longer overall processing texts, whereas momentary increases in enjoyment are associated with shorter early fixation durations, increased skipping, and a greater likelihood of rereading. Although the present design does not permit causal inference, the dissociation between within- and between-participant effects suggests that fluctuations in perceived value are systematically linked to dynamic adjustments in reading behaviour. Together, these findings are consistent with the view that perceptual, oculomotor, and linguistic processes during reading are closely coupled to the value readers assign to the materials being read.

## Supplementary information

Transparent Peer Review file

Supplementary Materials

## Data Availability

All book synopses and comprehension questions are publicly available: https://osf.io/2hx7w/files.Trial-level, word-level, and fixation-level reports are available as .csv files: https://osf.io/2hx7w/files. The materials and the data sets generated and analysed are available in the Open Science Framework (OSF) repository, 10.17605/OSF.IO/2HX7W. This repository also includes an R Markdown script that reproduces all analyses and generates the manuscript.

## Code availability

An R Markdown (.rmd) file is available to replicate all analyses and generate the original manuscript: https://osf.io/2hx7w/files.
