# Supplementary material for: Situational enjoyment is associated with gaze behaviour during reading
Source: Commun Psychol. 2026 May 13;4:109. doi: 10.1038/s44271-026-00472-1 (PMC13396464; doi:10.1038/s44271-026-00472-1)
Supplement: Supplementary file 2 — Supplementary Materials [file 44271_2026_472_MOESM2_ESM.pdf]

Situational enjoyment is associated with gaze behaviour during reading

Supplementary Materials

Adam James Parker, Amrita Bains, Dorothy Zhiyu Gao, Emma Hance, Yunxi Li, & Yifangjia Zhang

Supplementary Methods

Adult Motivation Reading Scale

Participants completed the Adult Motivation for Reading Scale[1] to assess trait-based reading motivation. Participants rated how much they agreed with 21 statements on a five-point Likert scale, from 1 (strongly disagree) to 5 (strongly agree). The 21 items cover four distinct subscales: reading as part of self (e.g., *Without reading, my life would not be the same*), reading efficacy (e.g., *If a book or article is interesting, I don't care how hard it is to read*), reading to do well in other realms (e.g., *If I am going to need information from material I read, I finish the reading well in advance of when I must know the material*), and reading for recognition (e.g., *It is important to me to get compliments for the knowledge I gather from reading*). The 21-item scale has good internal consistency (alpha = 0.85), and the subscales yield Cronbach's Alpha of 0.87, 0.72, 0.70, and 0.83, respectively.

Reading Engagement Questionnaire

Participants reported what they had read and how much time they spent reading one day prior to the experiment. They were given four options: 0–30 minutes, 30-60 minutes, 60-120 minutes, or more than 120 minutes.

Supplementary Results

Supplementary Pre-Registered Analyses

For the confirmatory analyses of decisions to wait and comprehension, we initially pre-registered a generalised linear mixed-effects analysis of trial-level data, but instead opted for an analysis where the effects of between-person (i.e., trait) and within-person (i.e., state-based situational/trial-level) reading enjoyment were disaggregated. In these Supplementary Materials, we report our original pre-registered analyses for complete transparency. The spread of enjoyment ratings per participant and item is shown in Supplementary Figure 1.

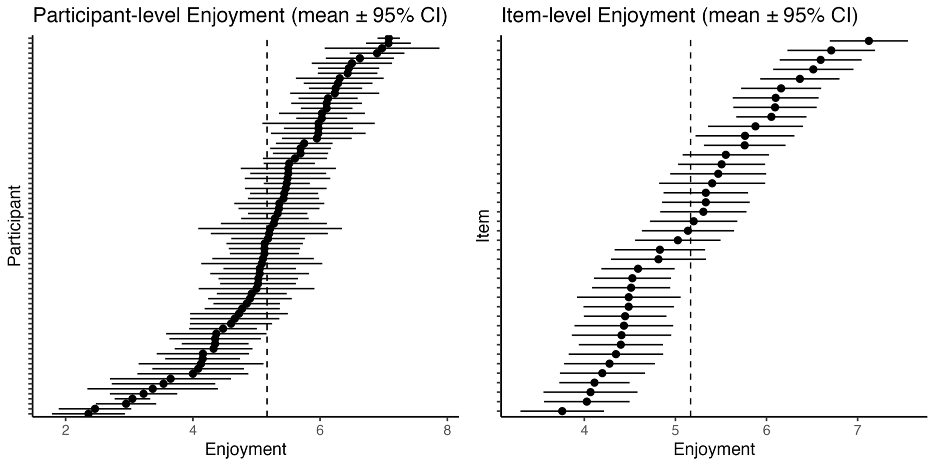

Supplementary Figure 1: **Participant- and item-level enjoyment.** Interval plot showing the mean enjoyment rating for each participant (76 participants) and item (40 items). The point represents the mean for each participant or item, and the error bar shows the 95% confidence interval around the mean.

Confirmatory Analyses The model fitted to binary decision to wait data (*glmer(dv~ centred reading enjoyment + (1 + centred reading enjoyment | participant) + (1 | item))*; 2,931 observations) indicated that centred reading enjoyment was a significant predictor of the likelihood of waiting to see the cover of a book (see Supplementary Table 1 and Supplementary Figure 2). That is, a one-unit increase in enjoyment was associated with a 2.55-fold increase in the odds of making a decision to wait.

The model fitted to binary comprehension accuracy data (*dv~ centred reading enjoyment + reading fluency + (1 | participant) + (1 | item)*; 5,862 observations) indicated that centred reading enjoyment was a significant predictor of the likelihood of answering a comprehension question correctly. As centred reading enjoyment increased, so did the likelihood to answer a question correctly, where a one-unit increase in enjoyment was associated with a 1.14-fold increase in the odds of answering a question correctly.

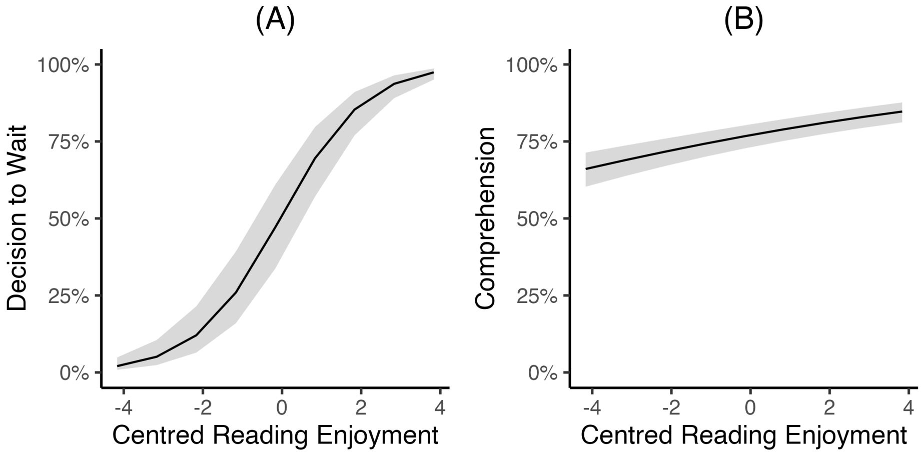

Supplementary Figure 2: **The Effect of Enjoyment on Decision to Wait and Comprehension.** The relationship between (A) trial-level centred mean enjoyment and decisions to wait, and (B) trial-level centred mean enjoyment and comprehension accuracy. Solid lines represent model-predicted probabilities from generalised linear mixed-effects models. Shaded bands indicate 95% confidence intervals around the fixed-effect estimates. Generalised linear mixed-effects models were fitted to 2,931 observations for the decision-to-wait analysis and 5,862 observations for the comprehension-accuracy analysis.

Supplementary Table 1: Generalized Linear Mixed-Effects Results for Comprehension and Decision to Wait.

Measure	Fixed Effect	b	SE	95% CI	z-value	p-value
Decision to Wait	(Intercept)	0.044	0.286	-0.516, 0.605	0.16	0.876
	Centred Reading Enjoyment	0.937	0.075	0.790, 1.085	12.46	< 0.001
Comprehension	(Intercept)	1.175	0.107	0.964, 1.385	10.95	< 0.001
	Centred Reading Enjoyment	0.131	0.017	0.097, 0.164	7.66	< 0.001
	Reading Fluency	0.033	0.006	0.021, 0.044	5.53	< 0.001

Exploratory Analyses

Global Reading Measures The model fitted to log-transformed reading time data (*lmer(dv~ centred reading enjoyment + reading fluency + passage difficulty + passage length + (1 + centred reading enjoyment | participant) + (1 + centred reading enjoyment | item))*; 2,931 observations) indicated that passage reading time increased as a function of trial-level reading enjoyment (see Supplementary Table 2 and Supplementary Figure 3). A one-unit change in enjoyment resulted in a 1.27% increase in passage reading time.

Supplementary Table 2: Linear Mixed-Effects Results for Passage Reading Time.

Measure	Fixed Effect	b	SE	95% CI	t-value	p-value
Passage Reading Time	(Intercept)	10.956	0.024	10.909, 11.002	463.63	< 0.001

Centred Reading Enjoyment	0.013	0.003	0.006, 0.019	3.65	< 0.001
Reading Fluency	-0.008	0.002	-0.012, -0.003	-3.53	< 0.001
Passage Difficulty	0.007	0.002	0.003, 0.012	3.06	0.004
Passage Length	0.004	0.000	0.004, 0.005	12.14	< 0.001

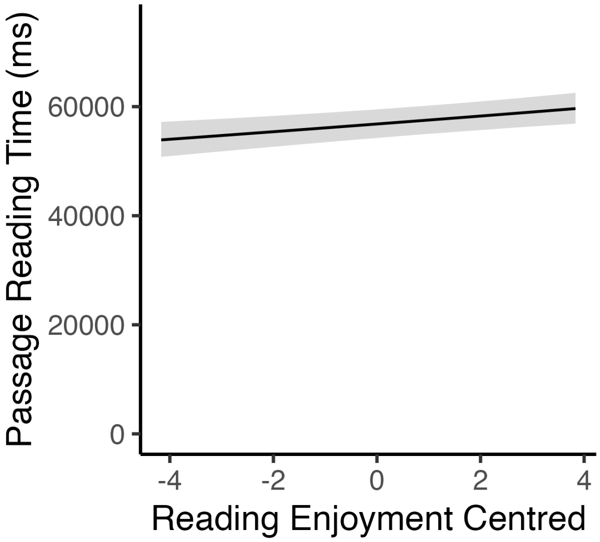

Supplementary Figure 3: **The Effect of Enjoyment on Passage-Level Reading Time.** The relationship between centred reading enjoyment and passage reading time. The solid line represents model-predicted values from linear mixed-effects models fitted to log-transformed passage durations. Shaded bands indicate 95% confidence intervals around the fixed-effect estimates. Linear mixed-effects models were fitted to 2,931 observations.

Local Reading Measures For local eye-movement measures, the final model included random intercepts only: *dv~ between-participant enjoyment + within-participant enjoyment + reading fluency + frequency + length + (1 | participant) + (1 | item) + (1 | word).* The model fitted to skipping likelihood data (210,277 observations) indicated that word skipping increased with increasing centred reading enjoyment (see Supplementary Table 3 and Supplementary Figure 4), where a one-unit increase in enjoyment was associated with a 1.01-fold increase in the odds of skipping a word. For first-fixation duration and gaze duration (139,680 observations each), there was a statistically significant effect of centred reading enjoyment. A one-unit change in enjoyment resulted in a 0.27% decrease in first-fixation duration and a 0.32% decrease in gaze duration, indicating that participants' initial reading times decreased as trial-level enjoyment increased. The model fitted to regression out likelihoods (210,277 observations) indicated that regressions increased with increasing centred reading enjoyment, where a one-unit increase in enjoyment was associated with a 1.01-fold increase in the odds of making a regression. This indicates that rereading increased as enjoyment increased. The model

fitted to total reading time data (210,277 observations) indicated that the estimate for within-participant enjoyment did not reach statistical significance for total reading time.

Supplementary Table 3: (Generalized) Linear Mixed-Effects Results for Word-Level Measures.

Measure	Fixed Effect	b	SE	95% CI	z/t-value	p-value
Skipping Likelihood	(Intercept)	-0.804	0.078	-0.956, -0.652	-10.37	< 0.001
	Reading Enjoyment Centred	0.013	0.003	0.008, 0.018	4.66	< 0.001
	Reading Fluency	0.011	0.008	-0.004, 0.026	1.41	0.159
	Zipf Frequency	0.001	0.015	-0.029, 0.031	0.04	0.965
	Word Length	-0.146	0.007	-0.160, -0.132	-21.19	< 0.001
First-Fixation Duration	(Intercept)	5.362	0.012	5.338, 5.386	433.05	< 0.001
	Reading Enjoyment Centred	-0.003	0.000	-0.004, -0.002	-5.60	< 0.001
	Reading Fluency	-0.006	0.001	-0.008, -0.003	-4.42	< 0.001
	Zipf Frequency	-0.031	0.002	-0.036, -0.027	-14.05	< 0.001
	Word Length	-0.001	0.001	-0.003, 0.001	-0.86	0.389
Gaze Duration	(Intercept)	5.439	0.014	5.411, 5.467	381.38	< 0.001
	Reading Enjoyment Centred	-0.003	0.001	-0.004, -0.002	-5.59	< 0.001
	Reading Fluency	-0.008	0.001	-0.011, -0.005	-5.61	< 0.001
	Zipf Frequency	-0.059	0.003	-0.065, -0.053	-19.89	< 0.001
	Word Length	0.018	0.001	0.015, 0.020	13.51	< 0.001
Regression Out Likelihood	(Intercept)	-1.864	0.045	-1.953, -1.776	-41.32	< 0.001
	Reading Enjoyment Centred	0.012	0.003	0.005, 0.019	3.46	< 0.001
	Reading Fluency	-0.002	0.004	-0.010, 0.005	-0.62	0.535
	Zipf Frequency	0.045	0.019	0.008, 0.082	2.38	0.017
	Word Length	0.093	0.008	0.077, 0.109	11.17	< 0.001
Total Reading Time	(Intercept)	5.694	0.020	5.655, 5.733	286.32	< 0.001
	Reading Enjoyment Centred	-0.001	0.001	-0.002, 0.001	-1.08	0.281
	Reading Fluency	-0.005	0.002	-0.009, -0.002	-2.82	0.006
	Zipf Frequency	-0.102	0.004	-0.111, -0.094	-24.30	< 0.001
	Word Length	0.025	0.002	0.022, 0.029	13.77	< 0.001

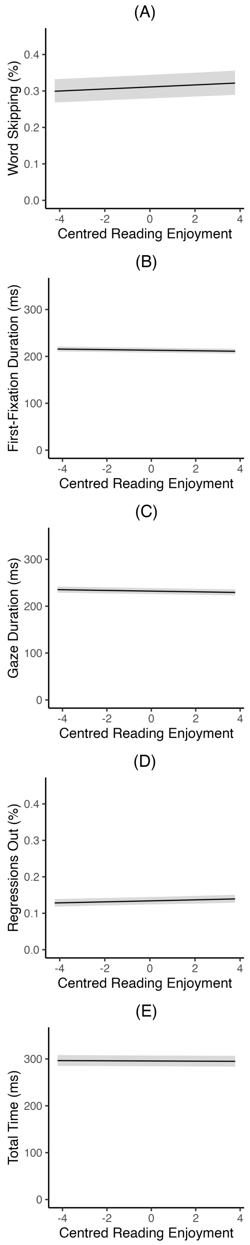

Supplementary Figure 4: **The Effect of Enjoyment on Word-Level Measures.** The relationship between centred reading enjoyment and (A) word skipping, (B) first-fixation duration, (C) gaze duration, (D) regressions out, and (E) total reading time. The solid line represents model-predicted values from (generalised) linear mixed-effects models fitted to binary outcomes (skipping and regressions out) and log-transformed reading times. Shaded bands indicate 95% confidence intervals around the fixed-effect estimates. Generalised mixed-effects models were fitted to 210,277 observations for skipping and regressions out. Linear mixed-effects models were fitted to 139,680 observations for first-fixation duration and gaze duration, and 201,277 observations for total reading time.

Summary Overall, the results from our pre-registered approach to the analysis indicate very similar patterns of results as the disaggregation approach reported in the main article. The same pattern of increased

decisions to wait and comprehension accuracy was observed when trial-level enjoyment increased. We also observed longer reading times overall at the passage-level that were accompanied by increased skipping rates, shorter initial reading times, and increased regressions back through the text.

Supplementary Exploratory Analyses of Text Properties

A substantial body of evidence demonstrates that readers' eye movements vary systematically as a function of text genre, including poetry, prose, and expository texts[2–6]. For example, narrative texts typically elicit more linear progression through the text, whereas expository and poetic texts are characterised by increased rereading and more frequent regressions[7]. Because genre is inherently linked to readers' subjective experience of a text, including enjoyment, it is therefore possible that the within-participant effects of enjoyment observed in our main analyses partially reflect broader, genre-level properties of the materials rather than enjoyment per se.

To evaluate this possibility, we repeated all analyses including a fixed effect coding mode of literature (fiction: -0.5; non-fiction: +0.5). We adopted this binary categorisation because the stimulus set comprised a roughly equal number of fiction (19) and non-fiction (21) texts, whereas a finer-grained genre classification would require modelling an eight-level factor with highly unequal cell sizes (Supplementary Table 4), substantially increasing model complexity and the risk of spurious effects.

Supplementary Table 4: Experimental Items by Genre.

Genre	Count
Fantasy & Mythology	2
Horror/Dark	5
Literary/Domestic Drama	4
Mystery/Crime/Thriller	4
Philosophy, Meaning & Spirituality	5
Politics, Justice & Social Systems	9
Science, Medicine & Nature	6
War, Conflict & Historical Trauma	5

The outcomes of the formal statistical models are shown in Supplementary Table 5. Across all analyses in which within-participant enjoyment was significant in the main models, these effects remained significant after including the fixed effect coding for whether the text was fiction or non-fiction. Taken together, these

results indicate that the effects of within-participant enjoyment cannot be attributed to broad genre-based differences in reading mode.

Supplementary Table 5: (Generalized) Linear Mixed-Effects Results for Exploratory Analyses of Text Properties

Measure	Fixed Effect	b	SE	95% CI	z/t-value	p-value
Decision to Wait	Intercept	-4.493	-1.745	1.402 , -7.241	-3.21	0.001
	Between-Participant Enjoyment	0.901	1.423	0.266 , 0.38	3.39	0.001
	Within-Participant Enjoyment	0.821	0.897	0.039 , 0.744	21.03	< 0.001
	Fiction	-0.614	-0.332	0.144 , -0.896	-4.27	< 0.001
	Within-Participant Enjoyment x Fiction	-0.033	0.096	0.066 , -0.162	-0.50	0.618
Comprehension	Intercept	0.075	0.676	0.306 , -0.525	0.25	0.806
	Between-Participant Enjoyment	0.215	0.325	0.056 , 0.104	3.80	< 0.001
	Within-Participant Enjoyment	0.121	0.156	0.018 , 0.086	6.76	< 0.001
	Fiction	-0.123	0.257	0.194 , -0.503	-0.64	0.526
	Reading Fluency	0.029	0.041	0.006 , 0.016	4.53	< 0.001
	Within-Participant Enjoyment x Fiction	0.030	0.102	0.036 , -0.041	0.83	0.404
Passage Reading Time	Intercept	10.485	10.699	0.109 , 10.272	96.11	< 0.001
	Between-Participant Enjoyment	0.090	0.131	0.021 , 0.049	4.30	< 0.001
	Within-Participant Enjoyment	0.013	0.019	0.003 , 0.006	3.64	< 0.001
	Fiction	-0.036	0.006	0.021 , -0.077	-1.68	0.101
	Reading Fluency	-0.010	-0.005	0.002 , -0.014	-4.13	< 0.001
	Passage Difficulty	0.009	0.014	0.002 , 0.004	3.53	0.001
	Passage Length	0.004	0.005	0.000 , 0.004	12.44	< 0.001
	Within-Participant Enjoyment x Fiction	-0.004	0.005	0.005 , -0.013	-0.83	0.414
Skipping Likelihood	Intercept	-0.753	-0.046	0.361 , -1.46	-2.09	0.037
	Between-Participant Enjoyment	0.002	0.137	0.069 , -0.134	0.02	0.981
	Within-Participant Enjoyment	0.012	0.018	0.003 , 0.007	4.40	< 0.001
	Reading Fluency	0.011	0.026	0.008 , -0.005	1.37	0.17
	Fiction	0.066	0.170	0.053 , -0.038	1.25	0.213
	Zipf Frequency	0.021	0.031	0.005 , 0.011	4.23	< 0.001
	Word Length	-0.131	-0.125	0.003 , -0.136	-46.15	< 0.001
	Within-Participant Enjoyment x Fiction	-0.006	-0.004	0.001 , -0.008	-5.99	< 0.001
First-Fixation Duration	Intercept	5.313	5.442	0.066 , 5.184	80.48	< 0.001
	Between-Participant Enjoyment	0.010	0.034	0.013 , -0.015	0.75	0.453
	Within-Participant Enjoyment	-0.003	-0.002	0.000 , -0.004	-5.62	< 0.001
	Reading Fluency	-0.006	0.004	0.005 , -0.016	-1.10	0.28

	Fiction	-0.006	-0.003	0.001 , -0.009	-4.44	< 0.001
	Zipf Frequency	-0.031	-0.027	0.002 , -0.036	-14.03	< 0.001
	Word Length	-0.001	0.001	0.001 , -0.003	-0.84	0.404
Gaze Duration	Within-Participant Enjoyment x Fiction	-0.002	0.000	0.001 , -0.004	-1.71	0.087
	Intercept	5.349	5.494	0.074 , 5.205	72.58	< 0.001
	Between-Participant Enjoyment	0.017	0.045	0.014 , -0.01	1.24	0.22
	Within-Participant Enjoyment	-0.003	-0.002	0.001 , -0.004	-5.61	< 0.001
	Reading Fluency	-0.007	0.008	0.008 , -0.021	-0.87	0.392
	Fiction	-0.009	-0.006	0.002 , -0.012	-5.77	< 0.001
	Zipf Frequency	-0.059	-0.053	0.003 , -0.065	-19.87	< 0.001
	Word Length	0.018	0.020	0.001 , 0.015	13.52	< 0.001
Regression Out Likelihood	Within-Participant Enjoyment x Fiction	-0.002	0.000	0.001 , -0.004	-1.65	0.099
	Intercept	-1.824	-1.437	0.197 , -2.21	-9.25	< 0.001
	Between-Participant Enjoyment	0.023	0.097	0.038 , -0.051	0.62	0.536
	Within-Participant Enjoyment	0.011	0.018	0.003 , 0.005	3.42	0.001
	Reading Fluency	-0.089	-0.038	0.026 , -0.14	-3.43	0.001
	Fiction	-0.003	0.005	0.004 , -0.011	-0.73	0.466
	Zipf Frequency	0.000	0.012	0.006 , -0.012	-0.02	0.985
	Word Length	0.047	0.054	0.003 , 0.041	14.71	< 0.001
	Within-Participant Enjoyment x Fiction	0.013	0.027	0.007 , 0.000	1.93	0.054
Total Time	Intercept	5.448	5.639	0.097 , 5.258	56.18	< 0.001
	Between-Participant Enjoyment	0.048	0.084	0.019 , 0.012	2.59	0.012
	Within-Participant Enjoyment	-0.001	0.000	0.001 , -0.002	-1.23	0.219
	Reading Fluency	-0.008	-0.004	0.002 , -0.012	-3.73	< 0.001
	Fiction	-0.030	-0.005	0.013 , -0.054	-2.38	0.022
	Zipf Frequency	-0.102	-0.094	0.004 , -0.111	-24.28	< 0.001
	Word Length	0.025	0.029	0.002 , 0.022	13.79	< 0.001
	Within-Participant Enjoyment x Fiction	0.000	0.001	0.000 , 0.000	0.40	0.691

81 Supplementary References

82 1. Schutte, N. S. & Malouff, J. M. Dimensions of reading motivation: Development of an adult reading motivation scale. *Reading Psychology* **28**, 469–489 (2007).

83 2. Blohm, S. *et al.* Reading poetry and prose: Eye movements and acoustic evidence. *Discourse Processes* **59**, 159–183 (2022).

84 3. Gómez-Merino, N., Fajardo, I., Ferrer, A. & Joseph, H. Eye movements of deaf students in expository versus narrative texts. *American Annals of the Deaf* **167**, 313–333 (2022).

85 4. Kaakinen, J. K. & Hyona, J. Perspective effects on expository text comprehension: Evidence from think-aloud protocols, eyetracking, and recall. *Discourse Processes* **40**, 239–257 (2005).

86 5. Kraal, A., Broek, P. W. van den, Koornneef, A. W., Ganushchak, L. Y. & Saab, N. Differences in text processing by low-and high-comprehending beginning readers of expository and narrative texts: Evidence from eye movements. *Learning and Individual Differences* **74**, 101752 (2019).

87 6. Magyari, L., Mangen, A., Kuzmičová, A., Jacobs, A. M. & Lüdtke, J. Eye movements and mental imagery during reading of literary texts with different narrative styles. *Journal of eye movement research* **13**, 1–35 (2020).

88 7. Markevich, M. & Streltsova, A. The influence of text genre on eye movement patterns during reading. *Journal of Eye Movement Research* **18**, 60 (2025).
