# Supplementary material for: Situational enjoyment is associated with gaze behaviour during reading
Source: Commun Psychol. 2026 May 13;4:109. doi: 10.1038/s44271-026-00472-1 (PMC13396464; doi:10.1038/s44271-026-00472-1)
Supplement: Supplementary file 1 — Transparent Peer Review file [file 44271_2026_472_MOESM1_ESM.pdf]

nature portfolio

Peer Review File

Situational enjoyment is associated with gaze behaviour during reading

Corresponding Author: Dr Adam Parker

This file contains all editorial decision letters in order by version, followed by all author rebuttals in order by version.

Version 0:

Decision Letter:

** Please ensure you delete the link to your author homepage in this e-mail if you wish to forward it to your coauthors **

Dear Dr Parker,

Thank you for your patience during the peer-review process. Your manuscript titled "Situational Enjoyment Influences Gaze Behaviour during Reading" has now been seen by 2 reviewers, and I include their comments at the end of this message. They find your work of interest but raised some important points. We are interested in the possibility of publishing your study in Communications Psychology, but would like to consider your responses to these concerns and assess a revised manuscript before we make a final decision on publication.

We therefore invite you to revise and resubmit your manuscript, along with a point-by-point response to the reviewers. Please highlight all changes in the manuscript text file.

Editorially, we consider that you should clarify the conceptual framing of key constructs, including describing the SVR task more precisely as a reading fluency measure and clearly specifying its role in the study. Given the correlational design and relatively small effect sizes, causal claims should be tempered, coefficients translated into interpretable units, and alternative explanations (e.g., fluent processing driving enjoyment, unmeasured text properties) more explicitly discussed, with attention to how the within/between dissociation informs, but does not resolve, directionality. The robustness of the analyses should be strengthened through sensitivity checks, including addressing the stimulus order coding error and providing justification or checks for the model selection strategy. Finally, we strongly encourage additional analyses or reporting that better link enjoyment, eye-movement measures, and comprehension, as well as brief documentation of the psychometric properties of the motivation scale.

As you revise the manuscript in response to these issues, please also implement all requests in the attached Mandatory Revision Requests document. All requirements listed in this document need to be fully met, or the work will be returned to you for further revisions without peer review. This workflow is in place to increase the likelihood that the paper will be accepted for publication. It reduces the number of rounds of revision (and review) and ensures that the reviewers vet a version of the article that is compliant with journal policies. If you have any questions regarding the required revisions, please contact the journal prior to resubmission to avoid a negative outcome.

We are committed to providing a fair and constructive peer-review process. Please don't hesitate to contact us if you wish to discuss the revision in more detail.

Please submit the following items:

- Revised manuscript
- Point-by-point response to the referees' comments
- Mandatory Revision Requests Table (attached).
- Cover letter (as a separate document)

via this link: Link Redacted .

** This url links to your confidential home page and associated information about manuscripts you may have submitted or are reviewing for us. If you wish to forward this email to co-authors, please delete the link to your homepage first **

We hope to receive your revised paper within 8 weeks; please let us know if you aren’t able to submit it within this time so that we can discuss how best to proceed. If we don’t hear from you, and the revision process takes significantly longer, we may close your file. In this event, we will still be happy to reconsider your paper at a later date, provided it still presents a significant contribution to the literature at that stage.

We look forward to seeing the revised manuscript and thank you for the opportunity to review your work.

Best regards,

Troby Lui, on behalf of

Yafeng Pan

Troby Lui, PhD
Associate Editor
Communications Psychology

Yafeng Pan, PhD
Editorial Board Member
Communications Psychology
orcid.org/0000-0002-5633-8313

REVIEWER EXPERTISE:
Reviewer #1: reading, motivation, eye-movement
Reviewer #2: reading, eye-movement

REVIEWER REPORTS:

Reviewer #1 (Remarks to the Author):

The manuscript addresses a relevant and timely subject that has not been sufficiently explored in the literature, contributing to an understanding of the role of motivation and enjoyment in reading processing, text comprehension, and oculomotor dynamics. The rigor of the methodology and statistical analysis cannot be overstated, particularly considering pre-registration and full data/code availability. I recommend publication after the following minor points have been addressed:

1) The SVR task would be better described as a reading fluency task than a general reading skill task, since it has more in common with traditional reading fluency tests (such as the Woodcock-Johnson reading fluency subtest) than reading comprehension tests (which focus on passage or text-level comprehension). In addition, its status as a control variable should be made clear, or its potential role should be discussed in the introduction

2) More information about the psychometric properties (such as internal consistency or factor structure) of the Adult Reading Motivation Scale should be provided, at least on the supplementary materials.

3) I would like to see some analysis of the potential link between enjoyment, comprehension, and eye-movement measures, which is considered in the discussion but not examined in the results. Despite the correlational nature of the findings, it would be interesting to explore potential mediations between enjoyment, specific eye-tracking measures (such as gaze duration, regressions, or total word reading times), and reading comprehension performance. This is not a requisite, but it would be strongly recommended to strengthen the discussion.

Reviewer #2 (Remarks to the Author):

The paper is well written. The structure is clear and logical, moving effectively from the gap in situational motivation research through methods to the key findings. Technical eye-movement terminology is introduced accessibly, and the disaggregation approach is explained clearly with appropriate citations. Tables are well-organized, and figures effectively visualize the between/within dissociations.

Minor issues: There are typographical errors that should be corrected (e.g., "siutational" on page 5, "ocuolomotor" on page 5). Some Discussion sentences are lengthy and could be streamlined. The footnote explaining why within-person enjoyment shows null effects on total reading time (faster first-pass reading balanced by increased regressions) contains important interpretive information that might warrant inclusion in the main text.

This manuscript investigates how reading enjoyment operates at both trait-like (between-person) and state-like (within-person) levels, examining how these components relate to engagement, comprehension, and eye-movement behavior. Seventy-six participants read 40 book synopses while eye movements were recorded, rated their enjoyment, answered comprehension questions, and decided whether to wait to view book covers. Using mixed-effects models that disaggregate within- and between-person enjoyment, the authors report that both components predict willingness-to-wait and comprehension. Crucially, only within-person enjoyment is associated with a distinctive eye-movement signature: shorter early fixation durations, increased skipping, and increased regressions. Between-person enjoyment shows a different pattern, predicting longer total reading times without affecting early measures. The authors interpret this dissociation as evidence that momentary intrinsic value dynamically tunes reading strategies.

The manuscript advances four claims: (1) within-person and between-person enjoyment should be disaggregated in reading motivation studies; (2) both predict willingness-to-wait and comprehension; (3) they produce qualitatively different eye-movement signatures; and (4) momentary motivation dynamically shapes cognitive resource allocation during reading.

The study disaggregates within- and between-person enjoyment in eye-movement research during naturalistic reading. The behavioral findings replicate Bains et al. (2023) and Jones et al. (2025), and the disaggregation approach follows established methodology (Wang & Maxwell, 2015). Prior work on motivation and eye movements (Rettig & Schiefele, 2023) used questionnaire measures without controlling for reading ability. The study demonstrates that state and trait enjoyment produce distinct processing signatures.

This work should interest reading researchers (methodological implications for measuring motivation), motivation researchers (behavioral evidence for Situated Expectancy-Value Theory), educational psychologists (implications for instruction), and eye-movement researchers (understanding top-down modulation of oculomotor control).

The core findings are convincing. Several features strengthen the conclusions:
The analyses control for reading ability, word frequency, word length, passage difficulty, and passage length. The within-person eye-movement pattern (faster early processing + increased regressions) is internally coherent—superficial processing would predict fewer regressions, not more. The dissociation between within-person effects (early measures) and between-person effects (total time only) argues against simple confound-based explanations; if easier texts drove both enjoyment and eye-movement patterns, both components should show similar effects. The behavioral results replicate prior work, and the sample size (76 participants, 40 items) provides reasonable power.

However, there are a few limitations that are tempering conclusions: The correlational design cannot establish that enjoyment causes strategy changes—fluent processing might cause enjoyment instead, though the between/within dissociation provides indirect evidence against this. Effect sizes are small (e.g., $b = -0.003$ for within-person enjoyment on log first-fixation duration) and their practical significance is unclear. Enjoyment may correlate with unmeasured text properties (genre, narrativity) that independently affect eye movements.

Further evidence that would strengthen conclusions:
- Experimental manipulation of enjoyment (e.g., inducing affect, manipulating choice)
- Mediation analyses testing whether regressions mediate the enjoyment-comprehension relationship
- Correlation between task-derived between-person enjoyment and the questionnaire-based Adult Reading Motivation Scale
- Replication with extended texts beyond short synopses

Overall, the demonstration that within-person and between-person enjoyment produce different patterns should encourage researchers to disaggregate these components; much prior work has conflated levels of analysis. The finding that momentary enjoyment links to a specific eye-movement signature bridges motivation research (typically focused on self-report) with mechanistic detail from eye-tracking. The paper fills an important gap and provides a template for integrating motivation and online processing measures.

The chosen mixed-effects models suit the hierarchical data structure. The disaggregation approach follows best practices. Covariates are well-chosen. Reporting of coefficients, standard errors, confidence intervals, and test statistics is adequate. However, it is important to note that
although the backward elimination via buildmer is a reasonable choice, it can inflate Type I error, and robustness checks with alternative random structures would strengthen confidence. Also,
coefficients on log-transformed outcomes could benefit from conversions to interpretable units (milliseconds, probability changes). The coding error related to the non-randomized stimulus order for 10 participants (13% of sample) could have benefited from sensitivity analysis and order effect checks.

Making all the supplementary materials publicly available is exemplary.

Improvement Suggestions:

- Directionality: Explicitly discuss how the between/within dissociation informs (without resolving) the causal question. The

dissociation is more consistent with a motivational account than pure processing-fluency confounding.

- Effect sizes: Provide interpretable estimates (e.g., "a 1-unit increase in within-person enjoyment corresponds to X ms decrease in first-fixation duration").

- Sensitivity analyses: Report whether excluding the 10 participants with non-randomized order changes conclusions, and whether order effects are evident in this subgroup.

- Text-level confounds: Clarify what mechanisms could plausibly produce spurious within-person effects given existing controls. If genre information is available, exploratory analyses would be informative.

- Theoretical alternatives: Briefly acknowledge alternative frameworks (interest theory, predictive processing accounts, cognitive load perspectives) and how future work might distinguish them.

Recommendation:
Accept with Some Small Revisions

The core findings are sound and make a meaningful contribution. The demonstration that within-person and between-person enjoyment produce qualitatively different eye-movement patterns has methodological and theoretical implications for both motivation and reading research. The transparency and reproducibility standards are exemplary. With revisions addressing effect size interpretation, sensitivity analyses for the coding error, and more nuanced discussion of directionality, the paper is suitable for publication.

* TRANSPARENT PEER REVIEW: Communications Psychology uses a transparent peer review system. This means that we publish the editorial decision letters including Reviewers' comments to the authors and the author rebuttal letters online as a supplementary peer review file. However, on author request, confidential information and data can be removed from the published reviewer reports and rebuttal letters prior to publication. If your manuscript has been previously reviewed at another journal, those Reviewers' comments would not form part of the published peer review file.

** Visit Nature Research's author and referees' website at www.nature.com/authors for information about policies, services and author benefits**

Communications Psychology is committed to improving transparency in authorship. As part of our efforts in this direction, we are now requesting that all authors identified as ‘corresponding author’ create and link their Open Researcher and Contributor Identifier (ORCID) with their account on the Manuscript Tracking System prior to acceptance. ORCID helps the scientific community achieve unambiguous attribution of all scholarly contributions. You can create and link your ORCID from the home page of the Manuscript Tracking System by clicking on ‘Modify my Springer Nature account’ and following the instructions in the link below. Please also inform all co-authors that they can add their ORCIDs to their accounts and that they must do so prior to acceptance.
https://www.springernature.com/gp/researchers/orcid/orcid-for-nature-research

For more information please visit http://www.springernature.com/orcid

If you experience problems in linking your ORCID, please contact the Platform Support Helpdesk.

Version 1:

Decision Letter:

** Please ensure you delete the link to your author homepage in this e-mail if you wish to forward it to your coauthors **

Dear Dr Parker,

Your manuscript titled "Situational Enjoyment Influences Gaze Behaviour during Reading" has now been seen by our reviewers, whose comments appear below. In light of their advice I am delighted to say that we are happy, in principle, to publish a suitably revised version in Communications Psychology.

We therefore invite you to revise your paper one last time to address the remaining concerns of our reviewers and a list of editorial requests. At the same time we ask that you edit your manuscript to comply with our format requirements and to maximise the accessibility and therefore the impact of your work.

EDITORIAL REQUESTS:

Please review our specific editorial comments and requests regarding your manuscript in the attached "Editorial Requests

Table". Please outline your response to each request in the right hand column. Please upload the completed table with your manuscript files as a Related Manuscript file.

If you have any questions or concerns about any of our requests, please do not hesitate to contact me.

SUBMISSION INFORMATION:

In order to accept your paper, we require the files listed here https://www.nature.com/documents/commsj-file-checklist.pdf .

OPEN ACCESS:

Communications Psychology is a fully open access journal. Articles are made freely accessible on publication. For further information about article processing charges, open access funding, and advice and support from Nature Research, please visit https://www.nature.com/commspsychol/open-access

At acceptance, you will be provided with instructions for completing the open access licence agreement on behalf of all authors. This grants us the necessary permissions to publish your paper. Additionally, you will be asked to declare that all required third party permissions have been obtained, and to provide billing information in order to pay the article-processing charge (APC).

* TRANSPARENT PEER REVIEW: Communications Psychology uses a transparent peer review system. On author request, confidential information and data can be removed from the published reviewer reports and rebuttal letters prior to publication. If you are concerned about the release of confidential data, please let us know specifically what information you would like to have removed. Please note that we cannot incorporate redactions for any other reasons.

* CODE AVAILABILITY: All Communications Psychology manuscripts must include a section titled "Code Availability" at the end of the methods section. We require that the custom analysis code supporting your conclusions is made available in a publicly accessible repository at this stage; please choose a repository that generates a digital object identifier (DOI) for the code; the link to the repository and the DOI must be included in the Code Availability statement. Publication as Supplementary Information will not suffice.

* DATA AVAILABILITY:
All Communications Psychology manuscripts must include a section titled "Data Availability" at the end of the Methods section. More information on this policy, is available in the Editorial Requests Table and at http://www.nature.com/authors/policies/data/data-availability-statements-data-citations.pdf.

Please use the following link to submit the above items:
Link Redacted
** This url links to your confidential home page and associated information about manuscripts you may have submitted or be reviewing for us. If you wish to forward this email to co-authors, please delete the link to your homepage first **

We hope to hear from you within two weeks; please let us know if you need more time.

Best regards,

Troby Lui

Troby Lui, PhD
Associate Editor
Communications Psychology

Yafeng Pan, PhD
Editorial Board Member
Communications Psychology
orcid.org/0000-0002-5633-8313

REVIEWERS' COMMENTS:

Reviewer #1 (Remarks to the Author):

This manuscript examines how reading enjoyment operates at both trait (between-person) and state (within-person) levels

and how these components relate to engagement, comprehension, and eye-movement behavior during naturalistic reading. The central claims—that trait and state enjoyment should be disaggregated, that both predict behavioral outcomes, and that they show distinct eye-movement signatures reflecting dynamic modulation of cognitive processing—are well motivated, novel, and likely to be of broad interest across psycholinguistics, educational psychology, and motivation research. The work is methodologically rigorous, with pre-registration, open data and materials, and appropriate use of mixed-effects modeling, including a well-justified disaggregation of within- and between-person effects and the inclusion of reading fluency as a covariate. The findings are generally convincing, particularly the dissociation between state-related early processing efficiency coupled with increased regressions and trait-related differences in overall reading time, which provides meaningful insight into how momentary intrinsic value may shape reading strategies. While the conclusions are largely supported by the data, some caution in interpretation would be appropriate given the correlational design, as causal direction cannot be definitively established, and the relatively small effect sizes would benefit from brief contextualization regarding their practical significance. The addition of mediation analyses is a valuable extension and helps clarify the relationship between enjoyment, eye movements, and comprehension, although their reporting and integration into the main text should be clearly visible and transparent. The statistical analyses are appropriate and thoroughly reported, and the manuscript provides sufficient methodological detail to enable full reproducibility. Overall, this is a strong, well-executed, and theoretically informative contribution that will likely influence future work on motivation and reading processes, and I recommend acceptance of the manuscript.

Reviewer #2 (Remarks to the Author):

Dear Mr. Parker and co-authors,

Thank you for your thorough and well-considered revisions to your manuscript.

Your revised manuscript is in good shape, and I am pleased to say that I am recommending the acceptance of your paper pending two minor corrections — a result-labeling error and a numeric inconsistency.

1. LABELING ERROR IN THE COMPREHENSION RESULTS

In the Results section, the reporting of the comprehension model contains what appears to be a copy-paste error that inverts the labeling of the between- and within-participant effects. Specifically, the sentence immediately following the introduction of the between-participant coefficient (b = 0.216, corresponding to a 1.24-fold increase in odds) reads: "indicating that participants with higher mean enjoyment ratings were more likely to answer a question correctly" — which is correct for the between-participant predictor. However, the next sentence then re-introduces within-participant enjoyment (b = 0.122, 1.13-fold increase) using similar language, creating ambiguity about which coefficient is being described at each point.

Please revise this paragraph to ensure that each odds ratio is clearly and unambiguously linked to its corresponding predictor (between-participant vs. within-participant enjoyment), so that readers can follow the results without cross-referencing the table.

2. INCONSISTENT OBSERVATION COUNTS ACROSS THE MANUSCRIPT

There is a discrepancy in the number of observations reported for the skipping and regressions-out models. Table 3 states these models were fitted to 210,227 observations, while the caption for Figure 3 reports 210,277 observations for the same models. The same figure caption also reports 201,277 observations for the total reading time model, which differs from the 210,227 figure used elsewhere.

Please review your analysis output and reconcile these counts consistently across the main text, tables, and figure captions. If any of these figures have changed as a result of the revisions, please also confirm that the model specifications reported in the text remain accurate.

I greatly enjoyed reading your manuscript.
Your study will make a wonderful contribution to the body of knowledge.
Thank you for caring!

** Visit Nature Research's author and referees' website at www.nature.com/authors for information about policies, services and author benefits**

Open Access This Peer Review File is licensed under a Creative Commons Attribution 4.0 International License, which permits use, sharing, adaptation, distribution and reproduction in any medium or format, as long as you give appropriate credit to the original author(s) and the source, provide a link to the Creative Commons license, and indicate if changes were made.
In cases where reviewers are anonymous, credit should be given to 'Anonymous Referee' and the source.
The images or other third party material in this Peer Review File are included in the article's Creative Commons license, unless indicated otherwise in a credit line to the material. If material is not included in the article's Creative Commons license and your intended use is not permitted by statutory regulation or exceeds the permitted use, you will need to obtain permission directly from the copyright holder.
To view a copy of this license, visit https://creativecommons.org/licenses/by/4.0/

Reviewer #1 (Remarks to the Author):

The manuscript addresses a relevant and timely subject that has not been sufficiently explored in the literature, contributing to an understanding of the role of motivation and enjoyment in reading processing, text comprehension, and oculomotor dynamics. The rigor of the methodology and statistical analysis cannot be overstated, particularly considering pre-registration and full data/code availability. I recommend publication after the following minor points have been addressed:

We thank Reviewer 1 for their positive review of our manuscript. It's always a pleasure to know that additional steps in the research workflow to promote openness and transparency have not gone unnoticed. We believe we have addressed each of your concerns and summarised our responses below.

R1.1. The SVR task would be better described as a reading fluency task than a general reading skill task, since it has more in common with traditional reading fluency tests (such as the Woodcock-Johnson reading fluency subtest) than reading comprehension tests (which focus on passage or text-level comprehension). In addition, its status as a control variable should be made clear, or its potential role should be discussed in the introduction

We thank Reviewer 1 for this clarification. We agree that the sentence verification task does not assess text- or discourse-level reading comprehension, and that it has more in common with established measures of reading fluency, where it indexes efficient sentence-level reading, drawing on decoding, lexical access, and basic semantic integration under time constraints. We, therefore, describe the sentence verification task as a measure of ***reading fluency*** in the revised manuscript.

We have also clarified the role of the sentence verification task in the study. Specifically, it was included as a control measure to ensure that the observed effects of enjoyment on eye-movement behaviour could not be attributed to basic reading fluency. This is now reflected on:

- In the introduction, we state: "*To ensure that the relationship between reading enjoyment and outcomes was not driven by baseline skill, we included reading fluency (measured via a sentence verification task) as a covariate in all analyses.*"
- In the methods, we state: "*The Sentence Verification Task (Garvin & Krishnan, 2021) was included as a control measure to index individual differences in sentence-level reading fluency. Reading fluency was controlled for in all analyses to ensure that observed effects of reading enjoyment on comprehension and eye-movement behaviour were not attributable to baseline differences in basic reading skill*."

which is in addition to the statement that "*reading fluency, as indexed by sentence verification score, was included as a covariate as…*", which originally appeared in the Data Analysis section of the original manuscript.

R1.2. More information about the psychometric properties (such as internal consistency or factor structure) of the Adult Reading Motivation Scale should be provided, at least on the supplementary materials.

*** Based on Reviewer 1's request, we have reported the internal consistency of the 21-item scale and each of the four subscales for the Adult Reading Motivation Scale in the supplementary materials, where the task was reported in our initial analysis.

R1.2. I would like to see some analysis of the potential link between enjoyment, comprehension, and eye-movement measures, which is considered in the discussion but not examined in the results. Despite the correlational nature of the findings, it would be interesting to explore potential mediations between enjoyment, specific eye-tracking measures (such as gaze duration, regressions, or total word reading times), and reading comprehension performance. This is not a requisite, but it would be strongly recommended to strengthen the discussion.

Both Reviewer 1 and Reviewer 2 noted that these analyses were useful in disentangling our suggestion that enjoyment may influence rereading, and that, in turn, affects comprehension. We have, therefore, conducted mediation analysis within a Bayesian framework in the revised manuscript to estimate the indirect effects. These analyses are fully reported in the revised manuscript and are not detailed here; however, they indicate that the eye-movement measures examined do not statistically mediate the enjoyment–comprehension relationship. Importantly, this finding does not diminish the value of the eye-movement analyses themselves. Rather, it clarifies that while situational enjoyment reliably modulates moment-to-moment reading behaviour, these changes do not account for the observed effect of enjoyment on comprehension accuracy. In the revised manuscript, we therefore emphasise that the eye-movement models provide critical insight into how enjoyment shapes online reading behaviour, while the mediation results suggest that enjoyment's contribution to comprehension likely operates through higher-level cognitive or motivational mechanisms beyond those captured by eye movements.

Reviewer #2 (Remarks to the Author):

The paper is well written. The structure is clear and logical, moving effectively from the gap in situational motivation research through methods to the key findings. Technical eye-movement terminology is introduced accessibly, and the disaggregation approach is explained clearly with appropriate citations. Tables are well-organized, and figures effectively visualize the between/within dissociations.

We thank Reviewer 2 for their time taken in reviewing our manuscript. The comments provided are both detailed and insightful. We have fully considered each point raised by Reviewer 2, and doing so will have undoubtedly strengthened the revised manuscript. We provide a point-by-point summary of how we address each point raised below, where our response is indicated with ***.

R2.1. Minor issues:

R2.1.1. There are typographical errors that should be corrected (e.g., "siutational" on page 5, "ocuolomotor" on page 5).
*** We thank Reviewer 2 for catching these typographical errors, and further went through the revised manuscript to correct minor issues such as these.

R2.1.2. Some Discussion sentences are lengthy and could be streamlined.
*** We have streamlined the longer sentences in the Discussion to improve clarity.

R2.1.3. The footnote explaining why within-person enjoyment shows null effects on total reading time (faster first-pass reading balanced by increased regressions) contains important interpretive information that might warrant inclusion in the main text.
*** We have included a discussion of this information in the main body of text in our revised manuscript.

R2.2. This manuscript investigates how reading enjoyment operates at both trait-like (between-person) and state-like (within-person) levels, examining how these components relate to engagement, comprehension, and eye-movement behavior. Seventy-six participants read 40 book synopses while eye movements were recorded, rated their enjoyment, answered comprehension questions, and decided whether to wait to view book covers. Using mixed-effects models that disaggregate within- and between-person enjoyment, the authors report that both components predict willingness-to-wait and comprehension. Crucially, only within-person enjoyment is associated with a distinctive eye-movement signature: shorter early fixation durations, increased skipping, and increased regressions. Between-person enjoyment shows a different pattern, predicting longer total reading times without affecting early measures. The authors interpret this dissociation as evidence that momentary intrinsic value dynamically tunes reading strategies.

The manuscript advances four claims: (1) within-person and between-person enjoyment should be disaggregated in reading motivation studies; (2) both predict willingness-to-wait and comprehension; (3) they produce qualitatively different eye-movement signatures; and (4) momentary motivation dynamically shapes cognitive resource allocation during reading.

The study disaggregates within- and between-person enjoyment in eye-movement research during naturalistic reading. The behavioral findings replicate Bains et al. (2023) and Jones et al. (2025), and the disaggregation approach follows established methodology (Wang & Maxwell, 2015). Prior work on motivation and eye movements (Rettig & Schiefele, 2023) used questionnaire measures without controlling for reading ability. The study demonstrates that state and trait enjoyment produce distinct processing signatures.

This work should interest reading researchers (methodological implications for measuring motivation), motivation researchers (behavioral evidence for Situated Expectancy-Value Theory), educational psychologists (implications for instruction), and eye-movement researchers (understanding top-down modulation of oculomotor control).

The core findings are convincing. Several features strengthen the conclusions:
The analyses control for reading ability, word frequency, word length, passage difficulty, and passage length. The within-person eye-movement pattern (faster early processing + increased regressions) is internally coherent—superficial processing would predict fewer regressions, not more. The dissociation between within-person effects (early measures) and between-person effects (total time only) argues against simple confound-based explanations; if easier texts drove both enjoyment and eye-movement patterns, both components should show similar effects. The behavioral results replicate prior work, and the sample size (76 participants, 40 items) provides reasonable power.

R2.3. However, there are a few limitations that are tempering conclusions: The correlational design cannot establish that enjoyment causes strategy changes—fluent processing might cause enjoyment instead, though the between/within dissociation provides indirect evidence against this. Effect sizes are small (e.g., $b = -0.003$ for within-person enjoyment on log first-fixation duration) and their practical significance is unclear. Enjoyment may correlate with unmeasured text properties (genre, narrativity) that independently affect eye movements.
*** We thank Reviewer 2 for highlighting these issues. Under **R2.6.4.**, we provide a detailed response to the comment about unmeasured properties of the text. Here we respond to the comment on tempering conclusions with regard to effect size. In our revised manuscript, we now add a statement in the discussion about the strength of effects.

Further evidence that would strengthen conclusions:

R2.3.1. Experimental manipulation of enjoyment (e.g., inducing affect, manipulating choice)
*** We agree with Reviewer 2 that this would provide a stronger test of the conclusions made, and we suggest this as a possible follow-up experiment.

R2.3.2. Mediation analyses testing whether regressions mediate the enjoyment-comprehension relationship
Both Reviewer 1 and Reviewer 2 noted that these analyses were useful in disentangling our suggestion that enjoyment may influence rereading, and that, in turn, affects comprehension. We have, therefore, conducted mediation analysis within a Bayesian framework in the revised manuscript to estimate the indirect effects. These analyses are fully reported in the revised

manuscript and are not detailed here; however, they indicate that the eye-movement measures examined do not statistically mediate the enjoyment–comprehension relationship. Importantly, this finding does not diminish the value of the eye-movement analyses themselves. Rather, it clarifies that while situational enjoyment reliably modulates moment-to-moment reading behaviour, these changes do not account for the observed effect of enjoyment on comprehension accuracy. In the revised manuscript, we therefore emphasise that the eye-movement models provide critical insight into how enjoyment shapes online reading behaviour, while the mediation results suggest that enjoyment's contribution to comprehension likely operates through higher-level cognitive or motivational mechanisms beyond those captured by eye movements.

R2.3.3. Correlation between task-derived between-person enjoyment and the questionnaire-based Adult Reading Motivation Scale
*** The correlation between between-participant enjoyment and the total score on the Adult Reading Motivation Scale was $r(74) = 0.16$, *95% CI* [–0.07, 0.37], $p = .169$. The size of the estimate is in keeping with prior research (Bains et al., 2023). Given that the measures obtained through the willingness-to-wait task exclusively index situational enjoyment during reading episodes, whereas the motivation scale reflects broader, self-reported motivational dispositions, such weak relationships are not surprising. Importantly, the between-participant enjoyment measure is not intended to capture trait-level reading enjoyment in the same sense as questionnaire-based measures, but rather stable individual differences in affective valuation of reading experiences across trials. We therefore interpret the weak association as reflecting a distinction between momentary enjoyment responses that are consistent within individuals and more global motivational constructs. We make note of this result in the revised manuscript via a footnote, where we summarise the utility of a behavioural between-participant measure of enjoyment that is not reducible to traditional questionnaire measures.

R2.3.4. Replication with extended texts beyond short synopses
*** We agree with Reviewer 2 that this would provide a stronger test of the conclusions made, and we suggest this as a possible follow-up experiment.

R2.4. Overall, the demonstration that within-person and between-person enjoyment produce different patterns should encourage researchers to disaggregate these components; much prior work has conflated levels of analysis. The finding that momentary enjoyment links to a specific eye-movement signature bridges motivation research (typically focused on self-report) with mechanistic detail from eye-tracking. The paper fills an important gap and provides a template for integrating motivation and online processing measures.

The chosen mixed-effects models suit the hierarchical data structure. The disaggregation approach follows best practices. Covariates are well-chosen. Reporting of coefficients, standard errors, confidence intervals, and test statistics is adequate. However, it is important to note that although the backward elimination via buildmer is a reasonable choice, it can inflate Type I error, and robustness checks with alternative random structures would strengthen confidence. Also, coefficients on log-transformed outcomes could benefit from conversions to interpretable units (milliseconds, probability changes). The coding error related to the non-randomized stimulus order

for 10 participants (13% of sample) could have benefited from sensitivity analysis and order effect checks.
*** Here, we specifically address the comment on random effects and the remaining points raised are addressed under **R2.6.2** and **R2.6.3**. Following Reviewer 2's recommendations, we refitted the models following the principled "keep it maximal" recommendations of Barr et al. (2013), with the fully random effects structure justified by the experimental design. We then implemented a principled, step-wise simplification procedure: switching optimiser, removing correlations, and removing the random effect with the least variance. Ultimately, this different approach yielded models with the same random effects structure, indicating that the data did not support random slopes of within-participant enjoyment for many of our dependent variables. We have added a footnote to the current manuscript to note this and, as the reviewer states, add confidence.

R2.5. Making all the supplementary materials publicly available is exemplary.
*** We thank Reviewer 2 for drawing attention to our efforts to make this work as transparent as possible.

R2.6. Improvement Suggestions:

R2.6.1. Directionality: Explicitly discuss how the between/within dissociation informs (without resolving) the causal question. The dissociation is more consistent with a motivational account than pure processing-fluency confounding.
*** In our revised manuscript, we have included this point in our discussion.

R2.6.2. Effect sizes: Provide interpretable estimates (e.g., "a 1-unit increase in within-person enjoyment corresponds to X ms decrease in first-fixation duration").
*** We thank Reviewer 2 for this suggestion, as it will undoubtedly aid the interpretation of our manuscript. We now report how a one-unit change in the predictor variable affected the outcome variable. For binary outcome measures, we report the odds ratio. For log-transformed reading time measures, we report the percentage change in duration associated with a one-unit change in the predictor to reflect that the coefficients on the log scale do not correspond to a constant ms change.

R2.6.3. Sensitivity analyses: Report whether excluding the 10 participants with non-randomized order changes conclusions, and whether order effects are evident in this subgroup.
*** Following Reviewer 2's recommendations, we conducted sensitivity analyses by removing the 10 participants with non-randomised items. Generally, the analyses yielded an identical pattern of results, and our conclusions remained unchanged. As a result, we have mentioned these sensitivity analyses in the footnote in the methods.

Regarding the analysis of order effects, we have carefully considered including a formal analysis in the revised manuscript. Still, given the sample size of this subset, we are concerned that such an analysis would lack sufficient statistical power to provide meaningful or reliable insights. In a sample of this size, individual variability can easily be mistaken for systematic order effects, potentially complicating the interpretation of the primary findings. We would like to

remind Reviewer 2 that the data have been made available via the OSF, allowing interested readers to conduct exploratory analyses without introducing underpowered inferential tests into the main text.

R2.6.4. Text-level confounds: Clarify what mechanisms could plausibly produce spurious within-person effects given existing controls. If genre information is available, exploratory analyses would be informative.
*** We thank Reviewer 2 for drawing our attention to the fact that potential mechanisms that could plausibly cause a spurious within-participant effect were not previously highlighted in our limitations. They are right to highlight how genre may have confounded our within-participant effect. We summarise the count of each genre below.

Genre	Count
Fantasy & Mythology	2
Horror/Dark Fiction	5
Literary/Domestic Drama	4
Mystery/Crime/Thriller	4
Philosophy, Meaning & Spirituality	5
Politics, Justice & Social Systems	9
Science, Medicine & Nature	6
War, Conflict & Historical Trauma	5

As can be seen, the current study includes a wide range of genres, and the distribution is relatively imbalanced. Because modelling this systematic imbalance of observations per genre, and the inclusion of a fixed effect resulting in seven pairwise comparisons, may lead to spurious results themselves, we have chosen not to examine this when revising the manuscript.

We have, however, taken this point on board and have run an analysis where we controlled for whether the synopses were fiction or non-fiction, where 19 were fiction and 21 were non-fiction. Across all analyses in which within-participant enjoyment was significant in the main models, these effects remained significant after including the fixed effect coding for whether the text was fiction or non-fiction. Taken together, these results indicate that the effects of within-participant enjoyment cannot be attributed to broad genre-based differences in reading mode. For completeness, these analyses are reported in the Supplemental Material and referenced in the limitations section of the manuscript.

R2.6.5. Theoretical alternatives: Briefly acknowledge alternative frameworks (interest theory, predictive processing accounts, cognitive load perspectives) and how future work might distinguish them.
*** In the revised Discussion, we now explicitly acknowledge complementary theoretical frameworks, including Situated Expectancy-Value Theory, Predictive Processing accounts, Cognitive Load Theory, and the Four-Phase Model of Interest Development. We outline how

each framework offers a distinct explanation for the observed eye-movement patterns. We note that future work is required to differentiate accounts.

R2.7. Recommendation:
Accept with Some Small Revisions

The core findings are sound and make a meaningful contribution. The demonstration that within-person and between-person enjoyment produce qualitatively different eye-movement patterns has methodological and theoretical implications for both motivation and reading research. The transparency and reproducibility standards are exemplary. With revisions addressing effect size interpretation, sensitivity analyses for the coding error, and more nuanced discussion of directionality, the paper is suitable for publication.
*** We again thank Reviewer 2 for their insightful comments and believe that we have addressed each comment above.

REVIEWERS' COMMENTS:

Reviewer #1

This manuscript examines how reading enjoyment operates at both trait (between-person) and state (within-person) levels and how these components relate to engagement, comprehension, and eye-movement behavior during naturalistic reading. The central claims—that trait and state enjoyment should be disaggregated, that both predict behavioral outcomes, and that they show distinct eye-movement signatures reflecting dynamic modulation of cognitive processing—are well motivated, novel, and likely to be of broad interest across psycholinguistics, educational psychology, and motivation research. The work is methodologically rigorous, with pre-registration, open data and materials, and appropriate use of mixed-effects modeling, including a well-justified disaggregation of within- and between-person effects and the inclusion of reading fluency as a covariate. The findings are generally convincing, particularly the dissociation between state-related early processing efficiency coupled with increased regressions and trait-related differences in overall reading time, which provides meaningful insight into how momentary intrinsic value may shape reading strategies. While the conclusions are largely supported by the data, some caution in interpretation would be appropriate given the correlational design, as causal direction cannot be definitively established, and the relatively small effect sizes would benefit from brief contextualization regarding their practical significance. The addition of mediation analyses is a valuable extension and helps clarify the relationship between enjoyment, eye movements, and comprehension, although their reporting and integration into the main text should be clearly visible and transparent. The statistical analyses are appropriate and thoroughly reported, and the manuscript provides sufficient methodological detail to enable full reproducibility. Overall, this is a strong, well-executed, and theoretically informative contribution that will likely influence future work on motivation and reading processes, and I recommend acceptance of the manuscript.

*** We thank Reviewer 1 for the thoughtful evaluation of our revised manuscript. Based on these, we have:

1. Carefully reviewed the manuscript and softened phrasing that could sound causal. This means that we have increasingly used “associated with” or “association” in the revised manuscript. This includes editing the title.
2. We now add an explicit comment in the discussion about practical significance before discussing implications of these results for intervention:

   Regardless, it is important to consider the practical significance of our results and any wider implications. Although the effects were modest in magnitude, this is not unexpected given the many factors that shape naturalistic reading. Their practical significance may therefore be incremental rather than large in isolation, but they nonetheless suggest that enjoyment is one factor that may meaningfully influence how readers allocate cognitive resources during comprehension. It is possible that such modest effects accumulate across paragraphs and become more consequential over longer texts, such as entire novels, such that enjoyment supports more sustained reading and improved comprehension.

3. We have attempted to make the exploratory analyses more visible and transparent in the revised manuscript. This was achieved by:
    a. Highlighting them from the outset of the manuscript by incorporating a short summary at the end of the introduction.
    b. Adding the following text to the discussion to integrate the analyses further:

        Accordingly, these exploratory analyses suggest that the association between situational enjoyment and comprehension remained after accounting for the aggregated trial-level reading measures examined here, rather than being statistically accounted for by them.

        We believe that this, accompanied by the existing paragraph in the Discussion, places the analyses more centrally in the revised manuscript.

Reviewer #2

Dear Mr. Parker and co-authors,

Thank you for your thorough and well-considered revisions to your manuscript.

Your revised manuscript is in good shape, and I am pleased to say that I am recommending the acceptance of your paper pending two minor corrections — a result-labeling error and a numeric inconsistency.

*** We thank Reviewer 2 for the time taken to read our revised manuscript, and are very appreciative of the fact that they spotted inconsistencies in our reporting.

R2.1. LABELING ERROR IN THE COMPREHENSION RESULTS

In the Results section, the reporting of the comprehension model contains what appears to be a copy-paste error that inverts the labeling of the between- and within-participant effects. Specifically, the sentence immediately following the introduction of the between-participant coefficient (b = 0.216, corresponding to a 1.24-fold increase in odds) reads: "indicating that participants with higher mean enjoyment ratings were more likely to answer a question correctly" — which is correct for the between-participant predictor. However, the next sentence then re-introduces within-participant enjoyment (b = 0.122, 1.13-fold increase) using similar language, creating ambiguity about which coefficient is being described at each point.

Please revise this paragraph to ensure that each odds ratio is clearly and unambiguously linked to its corresponding predictor (between-participant vs. within-participant enjoyment), so that readers can follow the results without cross-referencing the table.
*** We thank Reviewer 2 for bringing this to our attention. We have corrected the text in the revised manuscript.

R2.2. INCONSISTENT OBSERVATION COUNTS ACROSS THE MANUSCRIPT

There is a discrepancy in the number of observations reported for the skipping and regressions-out models. Table 3 states these models were fitted to 210,227 observations, while the caption for Figure 3 reports 210,277 observations for the same models. The same figure caption also reports 201,277 observations for the total reading time model, which differs from the 210,227 figure used elsewhere.

Please review your analysis output and reconcile these counts consistently across the main text, tables, and figure captions. If any of these figures have changed as a result of the revisions, please also confirm that the model specifications reported in the text remain accurate.

*** Again, we thank Reviewer 2 for bringing this to our attention. We can confirm that the models did not change in the main text as a result of revisions, and this appears to have been a copy-and-paste error. We have now updated these in the revised manuscript.

I greatly enjoyed reading your manuscript.
Your study will make a wonderful contribution to the body of knowledge.
Thank you for caring!
